# The Gut Microbiome in Enteric Viral Infections: Underlying Mechanisms and Therapeutic Approaches

**DOI:** 10.3390/microorganisms13102247

**Published:** 2025-09-25

**Authors:** Alejandro Borrego-Ruiz, Juan J. Borrego

**Affiliations:** 1 Departamento de Psicología Social y de las Organizaciones, Universidad Nacional de Educación a Distancia (UNED), 28040 Madrid, Spain; a.borrego@psi.uned.es; 2 Departamento de Microbiología, Universidad de Málaga, 29071 Málaga, Spain

**Keywords:** gut microbiome, gut microorganisms, gastrointestinal viral infections, molecular mechanisms, therapeutic tools, probiotics, fecal microbiota transplantation

## Abstract

Despite growing recognition of the role of the gut microbiome in host health and in modulating pathogen activity, the dynamic and reciprocal relationship between enteric viruses and the gut microbial ecosystem remains insufficiently defined and requires further exploration. This comprehensive review examines the bidirectional interplay between the gut microbiome and enteric viral infections by addressing (i) viruses associated with gastrointestinal alterations, (ii) how enteric viral infections alter the composition and function of the gut microbiome, (iii) how the gut microbiome modulates viral infectivity and host susceptibility, and (iv) current microbial-based approaches for preventing or treating enteric viral infections. Gastrointestinal viral infections induce gut microbiome dysbiosis, marked by reductions in beneficial bacteria and increases in potentially pathogenic populations. Specific gut microorganisms can modulate host susceptibility, with certain bacterial genera increasing or decreasing infection risk and disease severity. Pattern recognition receptors in the intestinal epithelium detect microbial signals and trigger antimicrobial peptides, mucus, and interferon responses to control viral replication while maintaining tolerance to commensal bacteria. The gut microbiome can indirectly facilitate viral infections by creating a tolerogenic environment, suppressing antiviral antibody responses, and modulating interferon signaling, or directly enhance viral replication by stabilizing virions, promoting host cell attachment, and facilitating coinfection and viral recombination. In turn, commensal gut bacteria can inhibit viral entry, enhance host antiviral responses, and strengthen mucosal barrier function, contributing to protection against gastrointestinal viral infections. Probiotics and fecal microbiota transplantation constitute potential microbial-based therapeutics that support antiviral defenses, preserve epithelial integrity, and restore microbial balance. In conclusion, the role of the gut microbiome in modulating enteric viral infections represents a promising area of future investigation. Therefore, integrating microbiome insights with virology and immunology could enable predictive and personalized strategies for prevention and treatment.

## 1. Introduction

The long-term coevolution between hosts and their commensal microorganisms has established a relationship of mutual benefit, largely mediated through the metabolic, physiological, and immunological functions of the microbiota [1]. While the terms microbiota and microbiome are sometimes used as equivalents, they describe distinct concepts. The microbiome refers not only to the diverse community of microorganisms, such as archaea, bacteria, fungi, protozoa, and viruses, present in a particular environment, but also to their collective genomes, structural components, metabolic products, and surrounding ecological context. In contrast, the term microbiota specifically denotes the populations of commensal, symbiotic, and pathogenic microorganisms inhabiting that environment [2]. Within the gastrointestinal (GI) tract, the whole microbial ecosystem is thus referred to as the gut microbiome (GM), which plays a central role in maintaining human health, including physiological, immune, metabolic, and mental domains [3]. Consequently, perturbations of the balanced state of the GM, a condition known as dysbiosis, can impair metabolic, immune, and neurocognitive functions, thereby contributing to a variety of diseases and health-related disorders. Moreover, dysbiosis compromises essential gut activities, including nutrient metabolism, pathogen defense, and immune regulation, ultimately affecting overall physiological homeostasis and health [4,5,6,7].

In recent years, recognition of the pivotal role of the GM in host health has greatly advanced our understanding of its interactions with invading pathogens. The GM contributes to host defense against infections through multiple direct and indirect pathways, including competition with pathogens for ecological niches and nutrients, secretion of antimicrobial compounds, and modulation of immune responses [8,9]. Among the pathogens that frequently colonize the GI tract, viruses are particularly common [10,11]. During infection, viruses inevitably encounter resident microbial communities in the gut, often engaging in complex interactions with them [10]. Evidence from numerous studies indicates that commensal microorganisms can influence viral invasion through diverse mechanisms, which may either limit or exacerbate disease outcomes [11,12]. Commensals, for instance, can suppress viral replication by reinforcing host defenses, while viral infection itself often disturbs microbial composition, causing dysbiosis that may, in turn, facilitate viral persistence or spread [13]. Increasing evidence further demonstrates that certain enteric viruses exploit gut microorganisms, particularly bacteria, to enhance their infectivity [14,15]. Indeed, members of different viral families have been observed to attach directly to bacteria or bacterial components, a process that promotes viral replication, pathogenesis, and transmission [16,17]. These findings challenge the traditional view of viral infection as a strictly cell-autonomous process, underscoring the importance of the surrounding microbial environment.

A variety of enteric viruses are recognized as major contributors to acute gastroenteritis. Among them, *Norovirus* (NoV) is responsible for nearly 20% of global cases [18], while *Rotavirus* (RV) remains a leading cause of infant mortality, particularly in low-resource settings [19]. RV primarily targets the epithelial lining of the small intestine, where it disrupts epithelial integrity and induces severe dehydration, accounting for a substantial proportion of deaths in children under five years of age in developing regions. Other viruses, including adenovirus, astrovirus, and *Sapovirus* (SaV), are also implicated in gastroenteritis outbreaks [20]. Transmission typically occurs via the fecal–oral route, most often through consumption of food or water contaminated with feces or vomit from infected individuals. Such infections are especially prevalent in areas with inadequate sanitation and limited access to clean water [21].

The bacterial fraction of the GM can act as a physical barrier against enteric viral infections within the intestine [17]. However, as previously noted, emerging evidence indicates that certain viruses are able to exploit these bacteria to enhance their own infectivity [22,23]. Supporting this notion, several preclinical studies have shown that antibiotic-mediated depletion of intestinal bacteria markedly shortens the duration of diarrhea and diminishes the infectivity of poliovirus and RV compared with untreated controls [24,25]. Notably, restoring the GM in antibiotic-treated mice was sufficient to re-establish viral pathogenesis [24,26]. In light of these findings, the GM has recently been recognized as a promising therapeutic target for combating enteric viral infections. Considerable progress has been achieved in the development of GM-based interventions, particularly through the use of probiotics and fecal microbiota transplantation (FMT). Clinical studies suggest that probiotics may help reduce the duration of viral gastroenteritis, alleviate symptoms, and shorten hospitalization time [27,28,29]. A meta-analysis of 19 randomized controlled trials (RCTs) further indicated that probiotic administration was associated with a lower risk of RV infection [30]. Nevertheless, evidence regarding their effectiveness remains inconsistent, as a review of probiotic use in RV-infected patients reported that only about half of the trials demonstrated clinical benefit, while the remainder found no significant effect [14]. At present, robust clinical RCT data supporting the use of FMT against enteric viral infections are lacking, with most findings limited to animal studies. Therefore, future research should focus on refining and standardizing these therapeutic approaches, while also advancing innovative strategies such as engineered probiotics, next-generation biotherapeutics, and precision microbiome-based interventions [31,32].

Despite growing recognition of the role of the GM in host health and in modulating pathogen activity, the dynamic and reciprocal relationship between enteric viruses and the gut microbial ecosystem remains insufficiently defined and requires further exploration, underscoring the need to synthesize current knowledge on the mechanisms underlying these interactions and their therapeutic implications. This comprehensive review examines the bidirectional interplay between the GM and enteric viral infections by addressing (i) viruses associated with GI alterations, (ii) how enteric viral infections alter the composition and function of the GM, (iii) how the GM modulates viral infectivity and host susceptibility, and (iv) current microbial-based approaches for preventing or treating enteric viral infections.

## 2. Viruses Associated with Gastrointestinal Alterations

A wide range of viruses are capable of infecting the GI tract, producing clinical outcomes that include watery or bloody diarrhea [33]. Beyond these typical manifestations, certain viruses are also associated with hepatitis, ulcerative conditions, motility disorders, and GI neoplasms, affecting both immunocompetent and immunocompromised individuals [34]. As outlined by Jagirdhar et al. [19], viral GI diseases can be broadly classified into three categories: (i) non-bloody diarrhea resulting from viral gastroenteritis, (ii) bloody diarrhea caused by viral gastroenteritis, and (iii) viral GI infections characterized by diarrhea accompanied by additional GI manifestations.

### 2.1. Viruses Associated with Non-Bloody Diarrhea

#### 2.1.1. Norovirus

NoV is a non-enveloped, positive-sense single-stranded RNA virus of the *Caliciviridae* family, subdivided into six genogroups and 49 genotypes [35]. According to the CDC, NoV represents the primary cause of acute gastroenteritis across all age groups in the United States [36]. Certain populations are at greater risk, including young children, the elderly, immunocompromised individuals, military personnel, and travelers. In the United States alone, NoV is estimated to result in approximately 109,000 hospitalizations annually. Outbreaks are most frequently reported in settings with close human contact, such as healthcare facilities, cruise ships, and restaurants, and transmission occurs predominantly via the fecal–oral route through contaminated food, water, or direct person-to-person spread [37]. Although NoV infection is usually self-limiting, with symptoms including diarrhea, nausea, vomiting, and abdominal cramping that typically resolve within two to four days, complications can occur. These include electrolyte imbalances, prolonged or chronic gastroenteritis, post-infectious irritable bowel syndrome (IBS), inflammatory bowel disease (IBD), and, particularly in children, neurological sequelae such as convulsions and encephalopathy [38]. Traditional methods for detecting NoV in clinical samples include electron microscopy, polymerase chain reaction (PCR), enzyme-linked immunosorbent assays (ELISAs), and immunochromatographic assays. More recently, advances in biosensor technology have introduced innovative detection platforms, including electrochemical, colorimetric, fluorescence-based, and CRISPR-driven biosensors [39]. Despite these diagnostic developments, no licensed vaccines against NoV are currently available. Ongoing clinical trials, however, are actively evaluating vaccine candidates for their potential to prevent infection and limit transmission.

#### 2.1.2. Rotavirus

RV is a double-stranded RNA virus within the *Reoviridae* family that represents the leading cause of diarrhea in children under five years of age. According to the Global Burden of Disease study, RV infection alone was responsible for 128,530 deaths in 2015, constituting 29.3% of all diarrheal fatalities that year [40]. Crawford et al. [41] highlighted that young children and individuals from lower socioeconomic backgrounds are particularly susceptible to severe disease. The diarrheal manifestations of RV infection arise from two primary mechanisms: (i) osmotic diarrhea resulting from malabsorption due to enterocyte damage, and (ii) secretory diarrhea mediated by activation of the enteric nervous system and the viral non-structural protein 4 [41]. Transmission occurs via the fecal–oral route or through contaminated surfaces (fomites) [37]. Clinically, RV infection presents with diarrhea, vomiting, and fever, with morbidity primarily driven by severe dehydration, which can necessitate hospitalization and, in some cases, lead to necrotizing enterocolitis [42]. Diagnostic approaches include ELISA, capable of detecting the virus up to one week after symptom onset, whereas real-time PCR (RT-PCR) provides higher sensitivity and can detect viral presence for extended periods [41]. The introduction of vaccines such as Rotarix, RotaTeq, Rotavac, and Rotasiil has substantially reduced the burden of RV disease, leading to a 45% decline in mortality among children under five since the mid-2000s [40,43].

#### 2.1.3. Astrovirus

Human astroviruses (HAstVs) are single-stranded RNA viruses classified within the genus *Mamastrovirus* of the *Astroviridae* family. Globally, they account for 0.5–15% of diarrheal outbreaks [44]. Transmission occurs primarily via the fecal–oral route, with fomites also serving as vectors [37]. Similar to other gastroenteritis-causing viruses, HAstV outbreaks are frequently reported in communal environments, including schools, nursing homes, and swimming pools [45]. The incubation period averages approximately 4.5 days, and infections generally present with mild diarrhea lasting 2–3 days, accompanied by fever, anorexia, and vomiting [46]. Diagnostic approaches include electron microscopy, cell culture, immunoassays, and RT-PCR, the latter being the most commonly employed technique [47]. The infection is typically self-limiting, with recovery supported by fluid and electrolyte replacement. However, prolonged illness and rare complications, such as meningitis and encephalitis, have been documented in immunocompromised adults and elderly patients [48].

#### 2.1.4. Sapovirus

SaV is a single-stranded positive-sense RNA virus within the *Caliciviridae* family. Rouhani et al. [49] reported that SaV represents the second most common cause of acute diarrhea after the bacterial pathogen *Shigella*, with an incidence of 22.8 cases per 100 child-years (95% CI: 18.9–27.5). Transmission occurs primarily via the fecal–oral route, as well as through contaminated food, water, and fomites [50]. SaV has been identified as a causative agent of gastroenteritis in both humans, especially children under five, and animals [51]. Outbreaks are frequently observed in communal settings, including daycare centers, hospitals, nursing homes, and schools [50,52]. Clinically, infection presents with symptoms similar to other viral gastroenteritis, although chronic diarrhea may occur in immunocompromised individuals [53]. Management of SaV-associated diarrhea generally involves rehydration, zinc supplementation, and maintenance of adequate nutrition [54]. Rarely, severe complications such as septic shock and intestinal obstruction have been documented [55,56].

#### 2.1.5. Enteroviruses

Enteroviruses are single-stranded positive-sense RNA viruses classified within the *Picornaviridae* family. The genus *Enterovirus* encompasses a wide array of viruses, including polioviruses, coxsackieviruses, echoviruses, rhinoviruses, and several other enterovirus subtypes [57]. A European surveillance study reported that between 2018 and 2023, approximately 563,654 enterovirus tests were performed, of which 33,265 (5.9%) were positive. In total, 11,605 cases were documented, covering 42 distinct virus types, with echoviruses 6, 9, 11, 18, and 30, coxsackieviruses A6, B4, and B5, and enteroviruses D68 and A71 being the most frequently detected [58]. Transmission primarily occurs via the fecal–oral route or through respiratory secretions, with infants, children, and adolescents showing higher susceptibility compared to adults [59]. After initial infection of the GI tract, these viruses can exhibit secondary tissue tropism, spreading to other organs and tissues [59]. Non-polio enteroviruses have been implicated in a variety of clinical conditions, including aseptic meningitis, hand, foot, and mouth disease (HFMD), myocarditis, pancreatitis, and flaccid paralysis [59,60]. GI symptoms, such as abdominal pain, vomiting, and diarrhea, are less common and generally self-limiting [60]. Severe complications, although rare, may include meningitis, encephalitis, myocarditis, and acute flaccid paralysis [61]. Enteroviruses can be detected in a variety of biological samples, including stool, pharyngeal swabs, blood, and cerebrospinal fluid, using methods such as PCR, serological assays, and cell culture techniques [62]. Several antiviral strategies are currently under investigation: (i) capsid-binding compounds and monoclonal antibodies, which inhibit viral attachment by blocking interactions between viral particles and host receptors; (ii) inhibitors of viral replication proteins, which interfere with polyprotein processing and the formation of replication organelles by targeting non-structural viral proteins; (iii) host factor inhibitors, which disrupt viral attachment and replication organelle biogenesis; and (iv) agents targeting RNA replication by modulating essential host pathways required for viral genome synthesis [63]. To date, the only licensed enterovirus vaccine is the poliovirus vaccine, which prevents poliomyelitis, although several other enteroviruses are under study as potential vaccine candidates [61].

#### 2.1.6. Human Adenoviruses

Human adenoviruses (HAdVs) are double-stranded DNA viruses belonging to the genus *Mastadenovirus* and *Adenoviridae* family. It has been established that HAdV-F40 and F41, classified as members of the F species, constitute the enteric serotypes of adenoviruses, which have been identified as the causative agents of acute gastroenteritis [64]. In infants, HAdV infections are considered as the second most frequent cause of diarrheal illness, just after RV [64]. The etiology of HAdV infections encompasses a broad spectrum of clinical manifestations, including febrile respiratory illness, pharyngoconjunctival fever, keratoconjunctivitis, hepatitis, and gastroenteritis [65]. The transmission of the pathogen occurs through various routes that include aerosols, fecal–oral contact, and fomites. Adenovirus infections manifest in settings characterized by close living and interaction, including daycare centers, summer camps, college campuses, and military camps [37,66]. The symptoms linked to adenoviral gastroenteritis are analogous to those observed in other forms of gastroenteritis, involving diarrhea, vomiting, and abdominal cramps. Nevertheless, a multicenter study demonstrated that fever was more frequently associated with adenoviral infection compared to other viral infections, excluding RV [67]. Complications of HAdV-related diseases include intussusception, hepatitis, chronic lung disease, meningoencephalitis, and cystitis [68,69]. At present, the FDA has approved the vaccination against adenovirus types 4 and 7 to prevent febrile acute respiratory disease for military populations aged 17 to 50 years [70].

#### 2.1.7. Coronaviruses

Coronaviruses are a group of single-stranded RNA viruses classified within the family *Coronaviridae*. The first recognized outbreak of a coronavirus, caused by severe acute respiratory syndrome coronavirus 1 (SARS-CoV-1), occurred between 2002 and 2003, affecting over 8000 individuals across 26 countries, with an estimated case fatality rate of approximately 10% [71]. Middle East respiratory syndrome coronavirus (MERS-CoV) emerged in 2012, causing sporadic outbreaks in the Middle East and other regions, with a reported case fatality rate of around 34% [72]. The emergence of severe acute respiratory syndrome coronavirus 2 (SARS-CoV-2) in late 2019 led to the coronavirus disease 2019 (COVID-19) pandemic, which has affected millions of people globally. By 2023, cumulative confirmed cases exceeded 435 million, with model-based estimates suggesting the actual number could surpass 773 million. Cumulative deaths exceeded 14.83 million, although vaccination campaigns are estimated to have prevented approximately 7 million deaths [73]. Coronaviruses are primarily transmitted via respiratory droplets, direct contact with infected individuals, and fomites [74]. Although human coronaviruses are primarily associated with respiratory infections, they have also been shown to induce GI symptoms, including diarrhea, nausea, vomiting, and abdominal pain. GI involvement is particularly noted in neonates, infants, and children, who are often co-infected with enteric viruses such as NoV and RV [75]. SARS-CoV-2 infection can manifest with GI symptoms that may occur independently of respiratory manifestations [76,77]. The pathophysiology of these GI symptoms is thought to involve the high expression of angiotensin-converting enzyme 2 (ACE2) receptors in the GI tract, which serve as the binding site for SARS-CoV-2 [78]. Evidence supporting fecal–oral transmission includes the detection of viral RNA in stool samples [75]. Currently, no specific antiviral therapies are approved for SARS-CoV-2 or MERS-CoV infections. Thus, treatment of COVID-19 relies on a combination of antiviral agents, neutralizing antibodies, Janus kinase inhibitors, and corticosteroids [79]. Several vaccines, including the Pfizer-BioNTech and Moderna mRNA vaccines, have been developed and report efficacies exceeding 90% in preventing symptomatic COVID-19 [80].

#### 2.1.8. Hepatitis E Virus

Hepatitis E virus (HEV) is a single-stranded RNA virus belonging to the genus *Orthohepevirus* within the family *Hepeviridae*. According to the WHO, approximately 20 million HEV infections occur globally each year [81], resulting in an estimated 44,000 deaths in 2015, accounting for 3.3% of viral hepatitis-related mortality [81]. HEV is primarily transmitted via contaminated food and water, but perinatal transmission and blood transfusions have also been documented [82]. Pregnant women and individuals with pre-existing liver disease are particularly vulnerable to severe HEV infection [83], while travelers to endemic regions, healthcare workers, and individuals consuming undercooked or raw pork may also be at increased risk [84]. Clinically, HEV infection is generally self-limiting, but patients may present with fever, anorexia, jaundice, nausea, vomiting, hepatomegaly, and abdominal pain [85]. Diagnosis relies on serological testing, enzyme immunoassays, or PCR-based RNA detection [86]. Acute hepatitis E typically does not require specific therapy. However, chronic HEV infection can be managed with a 12-week course of ribavirin [87]. Severe complications may include acute liver failure and neurological manifestations such as Guillain–Barré syndrome, myelitis, and neuropathy [88]. Currently, vaccines for HEV are not widely available, with the exception of those approved for use in China [89].

### 2.2. Viruses Associated with Bloody Diarrhea

#### 2.2.1. Cytomegalovirus

*Human cytomegalovirus* (HCMV) is a double-stranded DNA virus belonging to the *Herpesviridae* family. Its prevalence in the adult population ranges from 40% to 100% [90]. HCMV can remain latent and reactivate, particularly in immunocompromised individuals, such as transplant recipients, patients receiving immunosuppressive therapy, and individuals with IBD treated with corticosteroids [91]. Transmission occurs through contact with infectious body fluids, including saliva, urine, respiratory droplets, sexual contact, blood transfusions, and solid organ transplants [92]. Clinical manifestations of HCMV colitis vary widely and may include bloody diarrhea, abdominal pain, fever, weight loss, lymphadenopathy, as well as, in severe cases, toxic megacolon [93]. The severity of HCMV infection is influenced by age, with elderly patients at higher risk for complications such as toxic megacolon and pan-peritonitis [94]. Although HCMV reactivation is common in patients with IBD, studies have shown that it can spontaneously regress even without antiviral therapy, including cidofovir, foscarnet, ganciclovir, or valganciclovir [95,96].

#### 2.2.2. Herpes Simplex Virus

Herpes simplex virus (HSV) is a double-stranded DNA virus within the genus *Simplexvirus* and the *Herpesviridae* family. HSV proctitis represents the second most common sexually transmitted cause of infectious proctitis in men who have sex with men and may be caused by either HSV-1 or HSV-2, with approximately 70% of cases attributable to HSV-2 [97,98]. Transmission occurs through intimate person-to-person contact, including unprotected receptive anal or oral sex and sexual intercourse between men. Clinical manifestations of HSV proctitis include rectal bleeding, tenesmus, anorectal pain, and mucous discharge. In immunocompromised patients, HSV can lead to severe disseminated GI infections [99]. Prior to initiating immunosuppressive therapy for presumed IBD, it is pivotal to consider infectious proctitis, as immunosuppressants may fail to improve symptoms or exacerbate the infection. HSV-induced anogenital ulcers have been associated with a 1.5- to 7.0-fold increased risk of HIV transmission, likely due to disruption of the mucosal barrier [100]. Therefore, screening for HSV is essential. In individuals presenting with acute proctitis, particularly in the context of HIV infection or painful perianal ulcers, presumptive antiviral treatment should be considered. Confirmed or suspected HSV proctitis is managed with antiviral therapy using acyclovir, valacyclovir, or famciclovir [101]. Currently, no vaccines have been approved for the prevention of HSV infection.

### 2.3. Viruses Associated with Diarrhea and Other Gastrointestinal Manifestations

#### 2.3.1. Hepatitis A Virus

Hepatitis A virus (HAV), an RNA virus within the genus *Hepatovirus* of the *Picornaviridae* family, is responsible for a significant global burden of acute hepatitis. In 2017, an estimated 170 million cases of acute HAV infection were reported worldwide [102]. The virus is primarily transmitted via the fecal–oral route, most commonly through ingestion of contaminated food or water, although transmission through sexual contact, direct person-to-person exposure, and illicit drug use has also been documented [103]. Clinical presentation varies with age: over 70% of infections in children under six years remain asymptomatic, whereas approximately 70% of adults develop overt symptoms [104]. Manifestations of hepatitis A include fever, malaise, nausea, vomiting, abdominal pain, hepatomegaly, and jaundice [104]. The disease is typically self-limiting, but rare cases of relapsing hepatitis lasting up to one year have been reported [105]. Because of its usually benign course, specific treatment is generally not required. Diagnosis relies on serological testing, with IgM antibodies indicating recent infection, while IgG detection serves to assess immune status [106]. The Advisory Committee on Immunization Practices recommends routine HAV vaccination for all infants, as well as for subjects at high risk of exposure, those predisposed to severe hepatitis, individuals experiencing homelessness, and HIV-infected patients [107].

#### 2.3.2. Hepatitis B and D Viruses

Hepatitis B virus (HBV) is a partially double-stranded DNA virus classified within the genus *Orthohepadnavirus* of the *Hepadnaviridae* family. The CDC estimates that approximately 296 million individuals worldwide are living with HBV infection. Populations at increased risk include veterans, healthcare workers, men who have sex with men, individuals who inject drugs, and subjects co-infected with HIV or hepatitis C virus (HCV) [108]. Transmission occurs perinatally, sexually, via percutaneous exposure, or through direct contact with infected body fluids [109]. Following an incubation period of one to four months, acute HBV infection typically presents as a serum-sickness-like illness with fever, rash, and arthralgia, which may be followed by jaundice, nausea, vomiting, and other systemic symptoms [109]. Laboratory diagnosis relies on serum biomarkers, including elevated alanine aminotransferase, aspartate aminotransferase, and bilirubin [109]. Chronic HBV infection is a well-established risk factor for hepatocellular carcinoma (HCC) [110], which accounted for 80% of global liver cancer cases in 2018 [111]. The development of HBV-related HCC involves two principal mechanisms: (i) a direct oncogenic effect of viral proteins, which activate proto-oncogenes and signaling pathways such as MAP kinase and JAK/STAT, while inhibiting tumor suppressors like p53; and (ii) an indirect effect arising from chronic inflammation, cirrhosis, and hepatic regeneration [112]. Beyond HCC, HBV has been linked to other GI disorders. A pooled analysis of 702,754 individuals across 13 studies found that hepatitis B infection was associated with a 26-fold increased risk of gastric cancer, likely due to chronic inflammation, oncogenic viral proteins, and disruption of tumor suppressor pathways [113,114,115]. In addition, HBV infection has been associated with GM disturbances, particularly in patients with cirrhosis [116]. Acute HBV infection is typically self-limiting and does not require treatment. Management of chronic hepatitis B depends on factors such as cirrhosis status, alanine aminotransferase levels, and HBV DNA load, with therapeutic options including nucleos(t)ide analogues such as tenofovir and entecavir, or interferon (IFN)-based regimens [117]. Preventive strategies rely on recombinant HBV vaccines, administered in either two- or three-dose schedules [118].

Hepatitis D virus (HDV) is an RNA virus with defective replication that depends on co-infection with HBV for virion assembly and propagation. It is classified within the genus *Deltavirus* of the *Kolmioviridae* family. Globally, the burden of HDV/HBV co-infection is estimated to range between 62 and 72 million individuals [119]. While simultaneous HDV/HBV co-infection often resolves spontaneously [120], superinfection in patients with pre-existing HBV infection frequently progresses to chronic HDV infection, representing a particularly aggressive form of chronic hepatitis. Clinically, acute HDV infection presents similarly to other viral hepatitis forms. However, superinfection in HBV-infected individuals may precipitate fulminant hepatic failure [121]. Diagnosis relies on serological detection of anti-HDV IgM and IgG antibodies, with definitive confirmation obtained through HDV RNA testing [122]. Current therapeutic approaches primarily involve PEGylated-IFN administered for at least one year, although emerging treatments such as myrcludex B and lonafarnib have shown promising efficacy [123].

#### 2.3.3. Human Immunodeficiency Virus

Human immunodeficiency virus (HIV) is an RNA virus classified within the genus *Lentivirus* of the *Retroviridae* family. Recent global estimates indicate that approximately 1.3 million new HIV infections occurred in 2022 [124]. The virus is primarily transmitted through exposure to infected body fluids, including sexual contact, shared needles, breast milk, and perinatal routes. Beyond its systemic effects, HIV significantly impacts the GI system, a phenomenon specifically termed “HIV enteropathy” [125]. This condition is characterized by disruptions in epithelial ionic balance and induction of enterocyte apoptosis, leading to inflammation, altered intestinal permeability, and nutrient malabsorption. Histopathological changes include villous atrophy, crypt hyperplasia, and epithelial hyperproliferation, which collectively contribute to the development of diarrhea. In addition to enteropathy, HIV predisposes individuals to a spectrum of GI complications secondary to immunodeficiency. These may encompass esophageal disorders, gastritis, colitis, enteritis, and anorectal disease, often driven by opportunistic infections (bacterial, fungal, or viral) or HIV-associated neoplasms within the GI tract. The GI tract itself serves as a key site for viral replication and CD4^+^ T-cell depletion [126,127]. Extraintestinal manifestations may involve the pancreas and hepatobiliary system, manifesting as pancreatitis, exocrine pancreatic insufficiency, hepatitis, and metabolic dysfunction-associated steatotic liver disease (MASLD), which was formerly termed non-alcoholic fatty liver disease [128]. Antiretroviral therapy, particularly protease inhibitors, has also been linked to diarrhea in HIV-infected patients [129]. Diagnosis relies on fourth-generation antigen–antibody assays, followed by HIV-1 and HIV-2 differentiation testing [130]. Due to the high risk of opportunistic GI infections, stool examination for ova and parasites is recommended to identify pathogens such as *Cryptosporidium*, *Giardia*, and *Isospora* [131].

#### 2.3.4. Hepatitis C Virus

HCV is a single-stranded RNA virus with a primary tropism for the liver, classified within the genus *Hepacivirus* of the *Flaviviridae* family. Global prevalence estimates range between 0.5% and 2.5%, with the highest rates reported in the Eastern Mediterranean and European regions [132]. In 2016, HCV-related complications accounted for approximately 399,000 deaths worldwide [132]. A predictive model by Chen et al. [133] projected that the cumulative incidence of HCC in HCV-infected individuals could reach 583,000 cases between 2012 and 2040. Populations at elevated risk for HCV infection include injection drug users, HIV-positive individuals, healthcare workers, recipients of blood products, hemodialysis patients, sexual contacts of infected individuals, and neonates born to HCV-positive mothers [134,135]. Acute HCV infection is often asymptomatic, though some patients may experience fever, abdominal discomfort, and jaundice [135]. Spontaneous viral clearance occurs in only 15–20% of cases, with the majority progressing to chronic hepatitis C [135]. Chronic infection is defined by the persistence of HCV RNA beyond six months and can lead to significant liver pathology, including fibrosis, cirrhosis, and HCC [136]. Beyond hepatic manifestations, HCV has been linked to a spectrum of extrahepatic conditions that negatively impact quality of life, such as mixed cryoglobulinemia, glomerulonephritis, dermatologic disorders (e.g., porphyria cutanea tarda, lichen planus), and thyroid dysfunction (e.g., Hashimoto’s thyroiditis and Graves’ disease) [137]. GI symptoms associated with HCV include abdominal pain, nausea, vomiting, and alterations in bowel habits, reflecting liver injury and impaired bile secretion, which may compromise nutrient absorption and digestion. Furthermore, HCV infection has been associated with an increased risk of IBS and the development of esophageal and gastric varices secondary to portal hypertension [138]. Diagnosis relies on serological and molecular testing, including detection of anti-HCV antibodies and HCV RNA [139]. Current guidelines recommend early treatment with direct-acting antiviral agents following acute infection to reduce the risk of progression to chronic disease, given the high likelihood of asymptomatic persistence [140]. Chronic HCV is managed using pan-genotypic regimens such as sofosbuvir, velpatasvir, glecaprevir, and pibrentasvir, with therapy customized according to viral genotype and the severity of liver disease [140]. Although no vaccine is currently available, prevention focuses on minimizing exposure risks, including safe injection practices and adherence to safe sexual behaviors.

#### 2.3.5. Varicela-Zoster Virus

Varicella-zoster virus (VZV) is a double-stranded enveloped DNA virus, classified under the genus *Varicellovirus*, subfamily *Alphaherpesvirinae*, within the *Herpesviridae* family. Severe disease from primary VZV infection can occur in adults, young children, and immunocompromised individuals [141]. Prior to the introduction of vaccination programs in the United States, the annual incidence of VZV infections was estimated at approximately four million cases. Following widespread immunization, reported cases declined dramatically, with reductions as high as 97% [142]. VZV spreads through respiratory droplets, aerosols, or direct contact with infectious secretions or lesions from zoster [143]. Although GI involvement is rare, VZV has been reported to produce lesions throughout the stomach, duodenum, and small and large intestines [144]. In addition, isolated cases have documented constipation attributed to visceral neuropathy and motility disturbances [144,145]. Diagnosis is primarily guided by elevated liver enzyme levels and imaging abnormalities, and definitive confirmation can be achieved via immunohistochemical staining of tissue biopsies [146]. While clinical evaluation is typically sufficient for diagnosing shingles, PCR and immunoassays are often employed in atypical cases [147]. Management relies on antiviral therapy, with agents including acyclovir, valacyclovir, famciclovir, and brivudine [147]. Preventive strategies are supported by vaccination, with Varivax and Shingrix being approved for use [148].

#### 2.3.6. Epstein–Barr Virus

Epstein–Barr virus (EBV) is an enveloped double-stranded DNA virus classified within the genus *Lymphocryptovirus* of the *Herpesviridae* family. Globally, it is estimated that over 90% individuals experience EBV infection during their lifespan [149]. The virus is most commonly associated with infectious mononucleosis (IM), which primarily affects adolescents and young adults, presenting with fever, malaise, sore throat, and lymphadenopathy following transmission via saliva [150]. Chronic active EBV infection is defined by the persistence of IM symptoms beyond three months and is more frequently reported in Asian populations [151]. In addition to IM, EBV has been implicated in the pathogenesis of autoimmune conditions such as multiple sclerosis and rheumatoid arthritis [151]. Chronic EBV infection has also been linked to a spectrum of GI disorders, including gastritis, enteritis, esophageal disease, and oral hairy leukoplakia [152,153]. EBV is a recognized etiological factor in several lymphoproliferative malignancies, including Burkitt’s lymphoma, Hodgkin’s lymphoma, non-Hodgkin’s lymphoma, and post-transplant lymphoproliferative disorders [154]. Moreover, EBV has been associated with the development of non-lymphoid cancers, such as gastric carcinoma, nasopharyngeal carcinoma, and breast cancer [155]. Notably, EBV-related gastric cancers often localize to the gastric fundus, cardia, and body, distinguishing them from non-EBV-associated tumors, which more commonly arise in the antrum. Patients with IBD receiving immunosuppressive agents, such as thiopurines, show a high prevalence of EBV infection. Evidence indicate that thiopurine-treated individuals carry an increased risk of developing lymphoproliferative disorders compared to IBD patients not undergoing such therapy [156,157]. EBV diagnosis relies on the detection of antibodies against specific viral antigens, including viral capsid antigens, and can be established using several techniques, such as enzyme immunoassays, Western blot analysis, PCR-based assays, heterophile antibody agglutination tests, and other molecular methods [158]. Although in vitro studies have shown that antiviral agents such as acyclovir and cidofovir possess activity against EBV, their clinical efficacy has not been demonstrated [159].

#### 2.3.7. Human Herpesvirus 8

Human herpesvirus 8 (HHV-8), also referred to as Kaposi sarcoma-associated herpesvirus, is a double-stranded DNA virus classified within the genus *Rhadinovirus* of the *Herpesviridae* family [160]. Seroprevalence studies indicate that HHV-8 infection is most prevalent in Uganda, where Kaposi’s sarcoma (KS) is endemic [161], whereas in the United States, seroprevalence remains below 6% [162]. KS is a low-grade vascular neoplasm affecting both mucocutaneous and visceral sites, with frequent involvement of the respiratory and GI systems [161]. GI KS is often asymptomatic, although progressive disease may present with abdominal pain, nausea, vomiting, and GI bleeding [163]. Diagnosis is established via endoscopy and biopsy, with lesions most commonly found in the stomach and small intestine. Endoscopically, GI KS may appear as maculopapular lesions, nodular masses, or polypoid formations, which can occasionally bleed upon contact [164]. Treatment strategies vary based on disease extent. Localized KS may be managed with radiation therapy or intralesional chemotherapy, while systemic chemotherapy using agents such as liposomal doxorubicin or paclitaxel is reserved for more extensive involvement [165].

#### 2.3.8. Human Papillomavirus

Human papillomavirus (HPV) is a double-stranded DNA virus encompassing several genera, including *Alphapapillomavirus*, *Betapapillomavirus*, and *Gammapapillomavirus*, within the *Papillomaviridae* family. According to Lewis et al. [166], an estimated 13 million individuals in the United States acquired HPV in 2018, with over 77 million living with prevalent infection during the same year. HPV is primarily transmitted sexually via vaginal, anal, and oro-genital contact, although skin-to-skin and vertical transmission are also recognized routes [167]. Clinically, HPV manifests as cutaneous warts, anogenital warts, and respiratory papillomas, primarily caused by genotypes 6 and 11. Oncogenic genotypes, particularly 16 and 18, are implicated in precancerous lesions (intraepithelial neoplasias) and cancers [168]. HPV has been associated with multiple malignancies, including GI-related cancers such as esophageal, gastric, colorectal, anal, and hepatocellular cancers [169,170]. Cervical cancer is strongly linked to HPV infection at the transformation zone (squamocolumnar junction) [171], and anal cancers similarly arise at the squamocolumnar junction due to the presence of multipotent embryonic cells [172]. Diagnosis is achieved through colposcopy, biopsy, HPV DNA detection, PCR, and Pap smear testing [173]. Management depends on the lesion type. Warts may be treated with topical agents (e.g., salicylate, imiquimod, trichloroacetic acid), cryotherapy, or electrocautery, whereas precancerous or cancerous lesions require surgical excision and careful evaluation [174]. Vaccination remains a cornerstone of prevention, with FDA-approved bivalent (HPV 16 and 18), quadrivalent (HPV 6, 11, 16, 18), and 9-valent (HPV 6, 11, 16, 18, 31, 33, 45, 52, 58) vaccines protecting against genital warts, precancerous lesions of the cervix, vulva, and anus, as well as oropharyngeal cancers, and are approved for males and females aged 9–45 years [175].

## 3. Gastrointestinal Viral Infections and the Gut Microbiome

The GI tract harbors the most extensive and diverse population of commensal microorganisms in the human body. For this reason, the GM has become a central subject of scientific investigation [3,176], being now recognized as a key determinant of both health and disease, given its regulatory influence on essential physiological functions such as digestion, immune defense, and metabolic balance [177,178,179]. Disturbances in the composition or activity of the GM have been associated with multiple pathological outcomes, including the establishment of pathogenic species (among them viruses), heightened vulnerability to autoimmune conditions, metabolic disorders such as obesity, and GI diseases such as IBD [180,181,182].

### 3.1. Effects of Enteric Viral Infections on Gut Microbiome Composition

In mammals, intestinal microorganisms that contribute positively to host physiology are generally referred to as beneficial bacteria [183]. During viral infections, these protective populations often decline, while the abundance of detrimental species within the GI tract rises [184]. In a state of dysbiosis, both external pathogens and resident microorganisms can exploit the imbalance to colonize the host. A clear example is HIV infection, which compromises gut-associated lymphoid tissue and subsequently alters the composition of the GM. Within this context, members of the phylum Bacillota demonstrate the greatest transcriptional activity, supporting the maintenance of anti-inflammatory signaling pathways [185], although this shift is also linked to a reduction in the synthesis of short-chain fatty acids (SCFAs) and indole [186].

#### 3.1.1. Preclinical Studies

Several preclinical studies have examined how GI viral infections contribute to GM dysbiosis. One example is the transmissible gastroenteritis virus (TGEV), a member of the *Coronaviridae* family and the causative agent of transmissible gastroenteritis in pigs, characterized by severe diarrhea, vomiting, and dehydration [187]. Analysis of pigs infected with TGEV using RT-PCR revealed a marked reduction in *Lactobacillus* populations alongside an increase in *Enterobacteriaceae*, changes that may predispose animals to secondary bacterial infections [188]. Similar microbial alterations have been described in calves infected with RV, in which reductions in *Lactobacillus* coincided with an increase in *Escherichia* and *Streptococcus* [189]. In addition, Lei et al. [190] demonstrated that NoV infection significantly reshaped the intestinal microbiota of human GM-transplanted gnotobiotic (gn) pigs, affecting both phylum-level taxa (i.e., Bacillota, Bacteroidota, and Pseudomonadota) and various genera, including *Anaerococcus*, *Bacteroides*, *Bifidobacterium*, *Clostridium*, *Enterococcus*, *Lactobacillus*, and *Ruminococcus*. Evidence from animal models also suggests that SARS-CoV-2 infection induces gut dysbiosis, notably through the depletion of bacteria responsible for producing SCFAs in hamsters [191].

#### 3.1.2. Clinical Studies

Acute viral gastroenteritis (AGE) is a widespread condition most often caused by RV, NoV, HAstV, HAdV, and SaV. AGE remains a major contributor to illness and death worldwide, particularly among young children [192]. In one clinical investigation, fecal samples from 20 hospitalized children with severe or complicated AGE and 20 healthy controls were analyzed using 16S rRNA sequencing [193]. The authors reported that children with complicated AGE exhibited higher abundances of *Campylobacteriaceae*, *Neisseriaceae*, *Methylobacteriaceae*, *Sphingomonadaceae*, and *Enterobacteriaceae* compared with healthy counterparts. Similarly, Nelson et al. [194] explored whether NoV infection alters the GM by applying barcoded pyrosequencing of the 16S rRNA gene to stool samples from infected patients. Although the GM composition of most infected individuals was comparable to that of uninfected controls, a subset of patients displayed notable dysbiosis, marked by a reduction in Bacteroidota and an increase in Pseudomonadota. This latter increase was mainly attributable to the augmentation of *Escherichia coli*.

Mathew et al. [195] recently investigated the GM and clinical outcomes in young children affected by viral infections, specifically RV or NoV, alone or in combination with bacterial pathogens, specifically enteroaggregative *E. coli* (EAEC) and enteropathogenic *E. coli* (EPEC). Their findings revealed that children with viral–bacterial coinfections exhibited higher severity scores compared with those infected only by viruses. According to the Shannon diversity index, GM diversity fluctuated more substantially in the RV-infected cohort than in the NoV group. Notably, the abundance of *Bifidobacteriaceae*, a family with probiotic properties, increased in parallel with the severity of mixed viral–bacterial infections. As expected, both RV and NoV infections were linked to a reduction in *Bacteroides*, with the decrease being more pronounced when coinfection with *E. coli* was present. Among bacterial coinfections, EAEC produced more pronounced alterations in GM composition than EPEC alone, although both worsened clinical symptoms. Furthermore, increased prevalence of *Clostridiaceae* and *Streptococcaceae* was associated with aggravated vomiting and diarrhea in children infected with RV or NoV alone. A similar increase in *Streptococcaceae* was also observed in RV + EAEC coinfections. However, this enrichment diminished in RV + EPEC and RV + EPEC + EAEC triple infections. Differences between RV and NoV were also evident at the genus level, with *Prevotella* and *Ruminococcus* being more abundant in RV-infected children. The presence of these taxa in RV infection appears to be influenced by host factors, such as secretor status, and by viral genotype, particularly RV P-type 6 and P8 variants [196]. Moreover, EPEC coinfections were linked to increases in *Rothia*, *Leptotrichia*, and *Haemophilus*, along with reduced abundances of *Lactobacillus* and *Prevotella* in both RV and NoV cohorts. Conversely, EAEC coinfections were characterized by higher levels of *Oscillospira* and *Faecalibacterium*, as well as members of the *Lachnospiraceae* family. Triple infections involving RV + EAEC + EPEC were distinguished by an increase in *Collinsella*, *Roseburia*, and *Sutterella*.

Xiong et al. [197] carried out a prospective cohort study to compare the GM of infants with AGE caused by RV or NoV. Fecal samples were collected from 18 RV-infected infants (mean age 11.8 months) and 24 NoV-infected infants (mean age 8.8 months). Analysis of alpha diversity showed that the Chao1 index was significantly higher in the NoV group than in healthy controls, but lower in RV-infected infants when compared with NoV cases. No significant differences were observed in beta diversity between the two viral groups. Taxonomic profiling revealed that RV infection was associated with greater relative abundance of Actinomycetota at the phylum level and with increases in *Bifidobacterium*, *Streptococcus*, *Enterococcus*, and *Lactobacillus* at the genus level. In contrast, NoV infection was characterized by increased representation of Fusobacteriota and Cyanobacteriota, as well as higher levels of *Enterococcus* and *Streptococcus*. Comparative analysis showed that infants with RV infection exhibited higher abundance of Actinomycetota and Verrucomicrobiota, whereas Fusobacteriota predominated in NoV-infected infants. At the genus level, RV infection correlated with increases in *Veillonella* and *Bifidobacterium*, while NoV infection was linked to greater abundances of *Enterococcus*, *Clostridium*, and *Fusobacterium*. Linear discriminant analysis of effect size identified *Bacillus* as a characteristic genus in infants with viral gastroenteritis, both RV and NoV, followed by *Streptococcus* and *Enterococcus*. Furthermore, Random Forest analysis highlighted *Neisseria* as a marker distinguishing infants with viral diarrhea from healthy controls, whereas *Streptococcus* and *Pseudomonas* were discriminatory features separating healthy infants from those infected with RV and NoV, respectively.

Alterations in GM diversity have been documented immediately after SARS-CoV-2 infection [198,199,200,201]. Compared with healthy individuals, infected patients exhibited progressive changes in their GM beginning at disease onset, with the most pronounced alterations occurring within 2–3 weeks [199]. These shifts were largely driven by reductions in members of the families *Ruminococcaceae* and *Lachnospiraceae* [200], as well as in Bacillota, particularly the genus *Faecalibacterium*. In parallel, increases in opportunistic bacteria were also noted [198,201,202]. While most of these findings were reported in cohorts from China and Japan, studies in the United States revealed elevated levels of genera linked to GI disease, including *Campylobacter* and *Klebsiella*, specifically in African American patients [203].

Hepatitis virus infections have also been shown to impact GM diversity. For instance, one study reported increased GM diversity in treatment-naïve HCV patients [204], whereas another observed reduced alpha diversity in HCV-infected individuals [205]. Significant GM alterations have also been documented in HBV-related diseases. In patients with HBV-related acute-on-chronic liver failure (ACLF), genera such as *Veillonella*, *Streptococcus*, and *Enterococcus* were markedly increased, while HBV-related HCC patients displayed higher abundances of *Bacteroides*, *Lachnospiracea incertae sedis*, and *Clostridium* cluster XIVa [206,207,208]. Despite these observations, the relationship between GM changes and different types of viral hepatitis remains unclear. To address this, Yang et al. [209] reviewed 13 studies encompassing 950 individuals, including 656 patients (HBV, *n* = 546; HCV, *n* = 86; HEV, *n* = 24) and 294 healthy controls. The authors reported that GM diversity generally decreased with infection and disease progression. Specific taxa, including *Butyricimonas*, *Escherichia-Shigella*, *Lactobacillus*, and *Veillonella*, were identified as potential microbial markers for predicting hepatitis development. Among these, *Prevotella* was associated with reduced production of SCFAs, which are important for slowing HBV-HCC progression in HBx transgenic mice [210]. Conversely, *Lactobacillus*, *Escherichia-Shigella*, and *Veillonella* may contribute to pro-inflammatory factor production, including lipopolysaccharide (LPS) and tumor necrosis factor-alpha (TNF-α) [211]. In HCV-infected individuals, four genera (*Clostridia_UCG-014*, *Dorea*, *Monoglobus*, and *Ruminococcus*) were consistently reduced, suggesting potential roles in HCV prevention and therapy [209].

In addition, GM composition is altered in patients with acute hepatitis E (AHE) compared to healthy individuals, with certain bacteria within the phylum Pseudomonadota and the family *Enterobacteriaceae* correlating with IFN-γ levels [212]. Specifically, the presence of Pseudomonadia (formerly Gammaproteobacteria) showed positive associations with serum alanine transaminase and total bilirubin concentrations, indicating that these bacteria may serve as biomarkers for identifying AHE patients and predicting disease severity. Moreover, comparisons between patients with hepatitis E and those with HEV-induced acute liver failure revealed that fecal microbiota alterations were linked to HEV disease progression [213].

#### 3.1.3. Mendelian Randomization Studies

The interplay between viral infectious GI diseases (VIGDs) and GM dysbiosis has been documented, although the direction and causality of this relationship remain uncertain. Mendelian randomization (MR) is an analytical approach that leverages aggregated data from genome-wide association studies (GWASs) to investigate potential causal links between exposures and outcomes [214]. The principle underlying MR is that if a genetic variant influences the likelihood of a specific exposure or biomarker level and is also associated with a particular outcome, a causal effect of the exposure on the outcome can be inferred [215]. Compared to traditional observational studies, MR offers a robust method to minimize confounding factors, providing more reliable and precise estimates of causal effects. Furthermore, MR-derived causal estimates can be interpreted within a triangulation framework, which integrates evidence from complementary methodologies that rely on distinct assumptions [216].

Lyu et al. [217] applied MR to investigate associations between the GM and three common infections: intestinal infections, pneumonia, and urinary tract infections. Focusing on intestinal infections, they identified 14 GM features linked to disease susceptibility, spanning 1 phylum, 1 class, 2 orders, 2 families, and 8 genera. Protective associations were observed for class Deltaproteobacteria (OR = 0.026, 95% CI = 0.0046–0.14, *p* = 2.69 × 10^−5^), orders Desulfovibrionales (OR = 0.024, 95% CI = 0.0042–0.14, *p* = 2.55 × 10^−5^) and Enterobacteriales (OR = 0.024, 95% CI = 0.0042–0.14, *p* = 2.55 × 10^−5^), family *Enterobacteriaceae* (OR = 0.018, 95% CI = 0.0018–0.17, *p* = 5.37 × 10^−4^), and genera including *Alloprevotella* (OR = 0.17, 95% CI = 0.063–0.43, *p* = 1.87 × 10^−6^), *Erysipelotrichaceae* UCG003 (OR = 0.13, 95% CI = 0.054–0.33, *p* = 1.24 × 10^−5^), *Marvinbryantia* (OR = 0.019, 95% CI = 0.027–0.13, *p* = 6.18 × 10^−5^), *Ruminococcaceae* NK4A214 group (OR = 0.025, 95% CI = 0.004–0.14, *p* = 1.89 × 10^−6^), and *Subdoligranulum* (OR = 0.10, 95% CI = 0.036–0.30, *p* = 3.11 × 10^−5^). In contrast, increased risk of intestinal infection was linked to phylum Bacillota (OR = 3.73, 95% CI = 1.65–8.45, *p* = 1.60 × 10^−3^), family *Victivallaceae* (OR = 1.49, 95% CI = 1.17–1.90, *p* = 1.21 × 10^−3^), and genera including *Eubacterium ventriosum* (OR = 10.40, 95% CI = 3.18–34.06, *p* = 1.09 × 10^−4^), *Gordonibacter* (OR = 2.92, 95% CI = 1.79–4.74, *p* = 1.57 × 10^−5^), and *Collinsella* (OR = 3.25, 95% CI = 2.00–5.27, *p* = 1.87 × 10^−6^).

Song et al. [218] conducted a two-sample MR study using publicly available GWAS data to explore the relationship between the GM and COVID-19 outcomes. Using the Inverse Variance Weighted method, they identified 42 bacterial genera associated with COVID-19 susceptibility, hospitalization, and severity. Among these, five taxa showed significant associations with hospitalization and severe disease: phylum Actinomycetota (OR = 1.10, 95% CI = 1.03–1.18, *p* = 0.0021), order MollicutesRF9.id.11579 (OR = 1.13, 95% CI = 1.04–1.21, *p* = 0.0014), family unknownfamily [id.1000005471] (OR = 1.11, 95% CI = 1.02–1.22, *p* = 0.0698), genus unknowngenus [id.1000005472] (OR = 1.10, 95% CI = 1.02–1.17, *p* = 0.0019), and genus *Tyzzerella3* (OR = 0.95, 95% CI = 0.91–0.99, *p* = 0.0600). In addition, class Negativicutes (OR = 1.23, 95% CI = 1.11–1.37, *p* = 0.0009), class Actinobacteria (OR = 1.10, 95% CI = 1.03–1.18, *p* = 0.0021), and order Selenomonadales (OR = 1.13, 95% CI = 1.02–1.25, *p* = 0.0633) were linked to both COVID-19 susceptibility and hospitalization. In a complementary bidirectional MR study, Tian et al. [219] confirmed causal relationships between specific GM taxa and COVID-19 outcomes. The genus *Intestinimas.id.2062* (OR = 1.179, 95% CI = 1.006–1.383, *p* = 0.042) was associated with increased risk of severe COVID-19, whereas *Bifidobacterium.id.436* (OR = 1.126, 95% CI = 1.021–1.242, *p* = 0.017), *LachnospiraceaeUCG010.id.11330* (OR = 1.139, 95% CI = 1.009–1.287, *p* = 0.034), and *RikenellaceaeRC9gutgroup.id.11191* (OR = 1.081, 95% CI = 1.019–1.147, *p* = 0.009) were causally linked to higher risk of hospitalization due to COVID-19. Conversely, *RuminococcaceaeUCG014.id.11371* (OR = 0.822, 95% CI = 0.782–0.995, *p* = 0.042) appeared to confer a protective effect against hospitalization.

More recently, Hu et al. [220] applied MR to explore causal relationships between the GM and several VIGD, including HAdV, HCMV, EBV, and HSV-1. The study identified 7 GM taxa with positive causal effects and 13 taxa with negative effects across these four viruses. Specifically, six genera were linked to HAdV, five to HCMV, four to EBV, and five to HSV-1. For HAdV, MR analysis indicated that genetically predicted higher abundances of *Adlercreutzia* (OR = 1.229, 95% CI = 1.010–1.496, *p* = 0.040), *Lachnoclostridium* (OR = 1.379, 95% CI = 1.055–1.803, *p* = 0.019), *Lactobacillus* (OR = 1.229, 95% CI = 1.023–1.476, *p* = 0.027), and *Lachnospiraceae* ND3007 group (OR = 1.675, 95% CI = 1.042–2.693, *p* = 0.033) were associated with increased risk, whereas *Desulfovibrio* (OR = 0.753, 95% CI = 0.594–0.954, *p* = 0.019) and *Erysipelatoclostridium* (OR = 0.812, 95% CI = 0.679–0.972, *p* = 0.023) were protective. In HCMV, five genera (*Butyrivibrio*, *Ruminococcaceae UCG014*, *Terrisporobacter*, *Bilophila*, and *Turicibacter*) were linked to decreased risk (ORs 0.779–0.855, all *p* < 0.05). For EBV, higher predicted abundances of *Gordonibacter*, *Blautia*, *Veillonella*, and *Terrisporobacter* were associated with reduced risk (ORs 0.747–0.847, all *p* < 0.05). In the case of HSV-1, increased predicted levels of *Escherichia-Shigella* (OR = 1.165), *Oscillospira* (OR = 1.248), and *Eisenbergiella* (OR = 1.112) raised infection risk, whereas *Erysipelotrichaceae UCG003* (OR = 0.837) and *Anaerotruncus* (OR = 0.814) were associated with decreased risk.

## 4. Modulation of Enteric Viral Infections by the Gut Microbiome

Recent studies have highlighted that the outcomes of many pathogenic viral infections are strongly influenced by the GM of the host [221]. As noted by Huang [10] and Sarkar and Bhowmik [222], the influence of intestinal microorganisms on viral infections can be understood through multiple mechanisms. First, the GM can serve as a physical barrier, limiting viral access to host cells [223]. Second, it can activate protective immune responses against invading viruses. Third, certain interactions with the GM may directly enhance viral replication or infection [221]. Finally, the GM can indirectly facilitate viral infection through modulation of the host environment or immune signaling [11].

### 4.1. The Gut Microbiome as a Physical Barrier to Viral Attachment

The intestinal epithelium functions as the primary barrier between the gut lumen, containing the GM, and the underlying lamina propria and deeper tissues. This barrier is maintained by intestinal epithelial cells (IECs), mononuclear phagocytes (MNPs), and gut-associated lymphoid tissues (GALT), which collectively preserve spatial segregation, detect microbial signals, and modulate immune responses to prevent excessive inflammation [224]. Pattern-recognition receptors (PRRs), including membrane-bound Toll-like receptors (TLRs) and other host sensors, serve as the first line of detection for microbial signals in the GI tract, initiating innate immune defenses and promoting antigen-specific adaptive responses [225]. PRRs recognize conserved microbial, pathogen, and damage-associated molecular patterns, leading to canonical antimicrobial responses through the induction of inflammatory cytokines, chemokines, and IFNs. These responses further stimulate IECs to secrete antimicrobial peptides and mucus, and recruit and activate intestinal MNPs [226]. PRR signaling in the intestinal mucosa is tightly regulated to ensure effective defense against pathogens while maintaining tolerance to commensal microorganisms, thereby preventing intestinal pathology and preserving homeostasis [227]. Among the early responses triggered by PRRs is the production of IFNs, including types I, II, and III, which activate distinct yet overlapping signaling pathways to restrict viral replication. Type I IFNs generally mediate both local and systemic viral control and associated immune pathology, whereas type III IFNs primarily provide protection at mucosal surfaces [228].

### 4.2. The Gut Microbiome in the Intrinsic Immune Response to Enteric Viral Infections

The GM can indirectly facilitate enteric viral infections by modulating antiviral immune responses. For instance, the GM may create a tolerogenic environment that aids viral evasion, suppresses antiviral antibody production, or alters virus-induced IFN signaling [221]. Under steady-state conditions, IECs sense commensal bacteria through various innate immune receptors, leading to cytokine secretion that shapes immune responses and establishes a tolerogenic microenvironment within the gut [229]. Regulatory T cells (Treg cells) specific for commensal bacterial antigens are abundant in the intestine and help maintain immune tolerance toward the vast diversity of non-pathogenic microorganisms. However, the tolerogenic environment generated by IEC and Treg recognition of commensals could, in principle, modulate antiviral immunity [230]. Activated Treg cells can suppress other immune cell populations in an antigen-nonspecific manner, employing both contact-dependent mechanisms, such as engagement of co-stimulatory receptors on antigen-presenting cells, and contact-independent mechanisms, including secretion of immunoregulatory cytokines [231,232]. Consequently, the immune recognition of gut bacteria that are associated with enteric viruses may lead to bystander suppression of antiviral responses.

Indirect evidence indicates that immune recognition of commensal gut bacteria may lead to bystander suppression during NoV infection. In immunocompetent hosts, NoV infections typically elicit only mild inflammation [233,234]. However, infection of IL-10-deficient mice with murine NoV results in pronounced intestinal inflammation [235]. This inflammatory response was shown to be GM-dependent, as germ-free (GF) IL-10-deficient mice infected with the virus did not develop inflammation, while colonization of these GF mice with a defined GM restored virally induced intestinal inflammation [235]. Further research is needed to clarify how this GM-driven inflammatory response influences the control of acute viral infection and the establishment of adaptive immunity against the virus [221].

Although RV infectivity is reduced in the absence of gut microorganisms, antibiotic-treated mice exhibited substantially higher antiviral antibody responses compared to colonized controls [236]. This increase was observed for fecal IgA, serum IgA, and serum IgG levels 9–11 weeks post-infection, while antibody levels before 9 weeks were similar between groups. Consistent with these results, GF mice also showed enhanced serum antiviral antibody responses compared to conventionally housed animals. These findings suggest that commensal gut microorganisms can suppress the maintenance of antiviral antibody responses. Supporting this notion, antibiotic-treated mice displayed greater numbers of antibody-secreting cells in the intestinal lamina propria and Peyer’s patches 7 weeks post-infection, but not at 2 weeks, compared to controls [25]. Interestingly, in contrast to RV, antibiotic-treated mice infected with murine NoV showed reduced serum IgG levels 35 days post-infection in comparison to microbial-colonized mice [236], highlighting virus-specific differences in how gut bacteria influence antiviral immunity.

To investigate whether commensal bacteria enhance persistent murine NoV infection by modulating antiviral immunity, antibiotic-treated mouse strains deficient in specific immune components were infected with murine NoV [236]. This approach revealed that type I and type II IFN responses, as well as pattern recognition receptors TLR2, TLR4, and MDA5, adaptive immunity, and the autophagy pathway, were not required for bacterial regulation of viral persistence. In contrast, mice lacking the IFNλ receptor (type III IFN receptor), STAT1, a critical signaling molecule downstream of IFNλ, or IFN-regulatory factor 3 (IRF3), essential for IFNλ expression, all developed persistent NoV infection regardless of the presence of commensal gut bacteria. These findings support a model in which the GM suppresses production of IFNλ during NoV infection. This aligns with recent evidence demonstrating the essential role of type III IFN responses in preventing persistent NoV infection in the colon [237]. Notably, IFNλ acts on non-hematopoietic cells to block persistent infection, despite NoV exhibiting tropism for immune cells [26,237], suggesting an indirect mechanism of viral restriction. In addition, IFNλ controls RV infection in mice [238], raising the question of whether bacterial interactions similarly modulate IFNλ-mediated antiviral defense against other enteric viruses.

The role of lamina propria lymphocytes (LPLs) in controlling enteric viruses is well established. Mice lacking B and T cells develop chronic murine RV infections [239], and both antibody- and T cell-mediated responses are essential for the clearance of murine NoV [240]. Similarly, murine astrovirus persists in the absence of adaptive B and T cell immunity [241]. Although the GM can modulate LPL populations, potentially influencing adaptive antiviral responses, few studies have directly examined this link. Notably, antibiotic treatment has been shown to reduce murine RV infection by increasing the number of RV-specific IgA-secreting cells in the small intestine [25], implying that a diverse microbial community may have pro-viral effects by modulating adaptive immune targeting.

### 4.3. The Gut Microbiome in the Direct Facilitation of Enteric Viral Infections

The GM can directly promote the replication of enteric viruses through multiple mechanisms, such as stabilizing virions, which may increase viral transmissibility, and facilitating viral attachment to host cells. Evidence for GM-mediated enhancement of infection has been reported for several enteric viruses, including poliovirus, murine NoV, and coxsackievirus B3. In GF or bacteria-depleted animal models, these viruses exhibited reduced infectivity and/or pathogenicity [24,236,242], highlighting the broad significance of GM-viral interactions in determining infection outcomes.

Direct interactions between viruses and bacteria are complex and influenced by bacterial surface components, such as LPS in Gram-negative bacteria and peptidoglycan (PGN) across bacterial types, as well as viral capsid elements that mediate these interactions [226]. Enhanced viral infectivity following bacterial binding often results from increased viral stability. For instance, poliovirus engages LPS and PGN via its VP1 capsid protein, improving stability in the mouse gut and facilitating transmission between hosts [23]. Similarly, other picornaviruses interact with bacterial cells and LPS, which confers resistance to heat and bleach in vitro [243]. Coxsackievirus B3 binds Gram-negative bacteria through the O-antigen of LPS, increasing viral infectivity and stability in mice [243,244]. Reovirus, which causes intestinal inflammation in immunocompromised mice, directly interacts with LPS and PGN from diverse bacteria, enhancing thermostability in cell culture [16]. Multiple poliovirus particles can attach to a single bacterial cell, generating high local multiplicities of infection that promote coinfection of host cells and genomic recombination [245]. However, only certain bacterial strains support coinfection, likely due to their differential affinities for mammalian cells [22]. This bacterial facilitation of poliovirus coinfection can increase recombination rates nearly fivefold [22], and recombination among poliovirus strains, including vaccines, as well as with other enteric viruses, has been documented in humans [246,247], suggesting a role for bacterial interactions in viral evolution.

Enteric viruses may also bind bacterial products that mimic host attachment factors. NoV and RV attach to histo-blood group antigen-like (HBGA-like) glycoproteins on bacteria, which resemble HBGAs on human intestinal cells [248,249]. NoV binding to HBGA-like molecules on bacteria such as *E. coli*, *Enterobacter*, and *Clostridioides difficile* protects viral particles from heat stress and enhances infection of cultured cells [248,250]. Murine NoV binds both Gram-negative and Gram-positive bacteria, but only Gram-positive interactions increase thermostability without enhancing infectivity in vitro [251]. Conversely, some bacteria inhibit enteric viruses. For instance, *Limosilactobacillus* (formerly *Lactobacillus*) *reuteri* binds enterovirus 71 and coxsackieviruses A6 and A16, blocking viral entry [252]. *Candidatus Savagella* (formerly segmented filamentous bacteria) protects against murine RV via host-mediated effects, including altered gene expression, accelerated epithelial turnover, and direct viral neutralization [253]. LPS, PGN, and certain gut-associated bacteria also enhance HAstV thermostability and infectivity in Caco-2 cells [254], although donor-specific variability can confer either enhanced susceptibility or protection, suggesting that unidentified microbial factors may modulate HAstV infection [254].

### 4.4. The Gut Microbiome in the Indirect Promotion of Enteric Viral Infections

Indirect interactions between enteric viruses and the GM are mediated through viral-induced alterations of microbial composition and metabolism. Viruses influence the GM by modifying metabolic activities, leading to the production of diverse bioactive compounds that reshape the luminal chemical environment, affect host cell populations, and modulate immune responses. Many small metabolites generated by the GM can also circulate systemically, allowing the GM to impact viral infections at distal sites. Intestinal bacterial metabolism is largely driven by fermentation of undigested dietary carbohydrates and host-derived mucins. Notably, mucin degradation represents a key mechanism by which the GM modifies the intestinal environment and influences viral replication. During murine RV infection, increases in mucin-degrading bacteria such as *Bacteroides* and *Akkermansia* reduce the ability of RV virions to bind host cells both in vitro and in vivo [255].

Fermentation of carbohydrates by the GM generates SCFAs, which circulate through the bloodstream and influence both local and systemic immune responses. Many SCFAs act as histone deacetylase (HDAC) inhibitors, broadly modulating host epigenetic regulation and immune function [256], and can even activate latent viruses such as EBV [257]. Butyrate, which is a particularly potent HDAC inhibitor [258,259], has been shown to upregulate the coxsackievirus and adenovirus receptor (CAR) in colon cancer cell lines [260], suggesting that microbial-derived butyrate could indirectly increase susceptibility to HFMD caused by coxsackievirus A16 and enterovirus A71, both of which utilize CAR for cellular entry [261]. Another important microbial metabolite is succinate, produced during fermentation of carbohydrates and proteins. Succinate serves as a key cross-feeding metabolite within the intestinal ecosystem, typically maintained at low levels, but can accumulate during microbial dysbiosis caused by antibiotics or IBD [262]. This metabolite is sensed by succinate receptor 1, predominantly expressed on tuft cells, modulating mucosal immune responses and driving tuft cell proliferation [262,263]. Since tuft cells are targets for murine NoV [264], and RV [265], succinate production by the GM may enhance viral infection of these epithelial cells.

Protein metabolism by the GM generates a diverse array of bioactive compounds, including small metabolites and peptides derived from amino acid side chains. Fermentation of branched-chain amino acids such as valine, leucine, and isoleucine produces branched SCFAs, which in a way similar to butyrate act as potent HDAC inhibitors [266]. Moreover, metabolites resulting from microbial amino acid processing influence both innate and adaptive immune responses [267]. These protein- and amino acid-derived metabolites represent a key pathway of chemical communication between the GM and the host immune system, potentially modulating susceptibility to viral infections.

**Figure 1:**
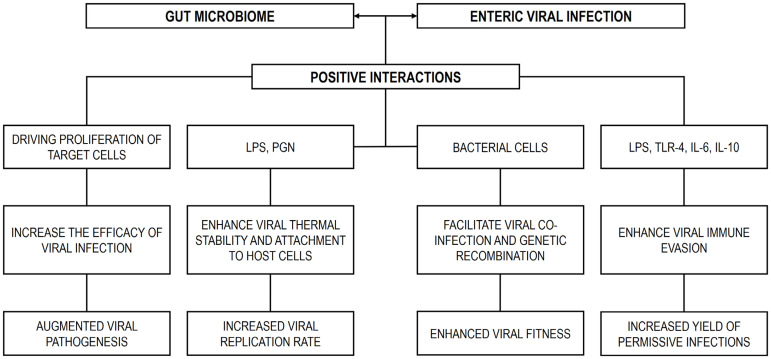
Mechanisms by which the GM positively interacts with enteric viral infections. LPS: lipopolysaccharides; PGN: peptidoglycan; TLR-4: Toll-like receptor 4.

**Figure 2:**
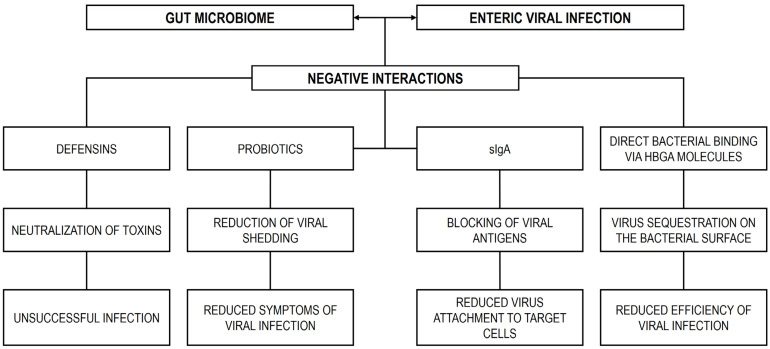
Mechanisms by which the GM negatively interacts with enteric viral infections. sIgA: secretory immunoglobulin A; HBGA: histo-blood group antigens.

Bile acids (BAs), synthesized from cholesterol in the liver, are secreted into the intestinal lumen following food intake to aid lipid solubilization. In humans, primary BAs are predominantly chenodeoxycholic acid (CDCA), which can also be conjugated with glycine or taurine to form glycochenodeoxycholic acid (GCDCA) and taurochenodeoxycholic acid. Many intestinal bacteria express bile salt hydrolases that deconjugate taurine- or glycine-bound BAs back to their primary forms. This bacterial-mediated deconjugation has three major consequences: (i) it enhances BA tolerance for the microbiota, (ii) it promotes BA reabsorption and enterohepatic recycling in the host, and (iii) it provides primary BAs for further microbial modification into secondary BAs such as lithocholic acid (LCA) and deoxycholic acid (DCA) [226]. The GM, therefore, plays a pivotal role in shaping the luminal BA pool, which can modulate virus–host interactions. Murine NoV, for instance, utilizes GCDCA and LCA as cofactors for receptor binding [268]. Similarly, porcine enteric calicivirus depends on BAs for cell entry, endosomal escape, and modulation of antiviral IFN responses [269,270]. In contrast, RV replication is inhibited by CDCA and DCA through activation of the farnesoid X receptor (FXR), a key BA sensor regulating cholesterol, BA, and lipid homeostasis, potentially via downregulation of lipid synthesis [271]. Beyond local effects, the BA pool and FXR signaling may also exert systemic influence on viral infections, as observed for hepatitis C virus [272]. Figure 1 and Figure 2 provide an overview of the mechanisms by which the GM interacts with enteric viral infections, illustrating both direct and indirect effects (modified from [10,13,14]).

## 5. Microbial Treatment of Gastrointestinal Viral Pathogens

As previously stated, the GM, composed of commensal microorganisms, their metabolites, and associated microbial genes, establishes a dynamic network with both the host and invading viral pathogens, thereby shaping the outcome of infectious diseases through multiple mechanisms. On one hand, microbial populations and their metabolic products can act directly on pathogens by influencing their ability to colonize and their virulence potential. On the other hand, the GM interacts closely with the host across several regulatory layers, including immune and inflammatory pathways, metabolic and neuroendocrine functions, genetic background, circadian rhythms, and aging, primarily via the gut–brain axis [273].

Accumulating evidence indicates that viral infections of the GI tract are strongly linked to disruptions and imbalances in the GM. Clinical studies have shown that administering probiotics orally during the acute phase of illness can accelerate recovery and lessen the severity of GI disorders [274,275]. Moreover, both the composition and diversity of the intestinal microbial community have been associated with the effectiveness of vaccines targeting enteric viruses, including RV [276] and human norovirus (HNoV) [277]. These observations highlight the need to further investigate the interactions between the host and its microbiota, as well as between enteric pathogens and the intestinal microbial ecosystem.

### 5.1. Antiviral Effects of Probiotics

Multiple studies have demonstrated that probiotics, as well as their metabolites, can lower the risk of enteric viral infections and counteract viral interference with host homeostasis. Several mechanisms have been proposed to explain how probiotic bacteria exert these antiviral effects. One route involves direct viral antagonism through long-term evolutionary adaptations [183]. For instance, bacteria have developed defense strategies such as the CRISPR/Cas system, which serves as an intrinsic antiviral mechanism [278]. Beyond these direct interactions, probiotics and their metabolic products can also modulate host immunity, enhancing both innate and adaptive responses to limit viral infection [33,279].

**Figure 3:**
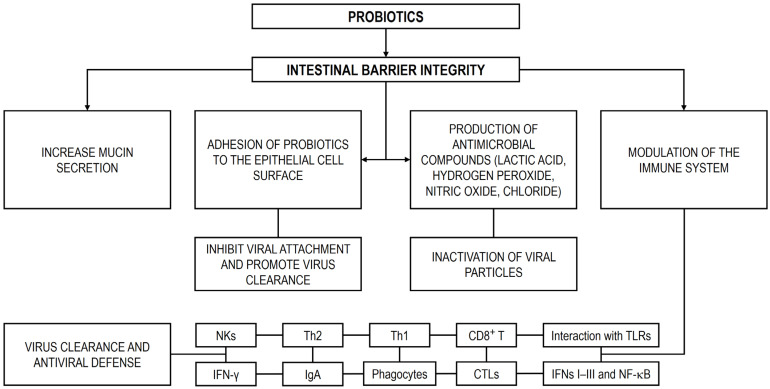
The impact of probiotics against viral infections is based on the probiotic–microbiota crosstalk with the aim of maintaining the integrity of the intestinal barrier by (i) promotion of mucin secretion; (ii) adhesion and binding of probiotics to the epithelial cell surface with the aim of blocking viral attachment either by steric hindrance, covering receptor sites in a non-specific manner, or competing for specific carbohydrate receptors; (iii) production of antimicrobial compounds such as lactic acid, hydrogen peroxide, nitric oxide, chloride ions; and (iv) modulation of the immune system. In the case of the immune system these include the following: (a) activation of natural killer cells (NKs), resulting in IFN-γ expression and activation of antiviral defense; (b) type 2 T-helper cells (Th2) capable of producing high levels of IgA; (c) type 1 T-helper cells (Th1) that will activate phagocytes and promotes virus clearance; (d) upon activation, CD8^+^ T lymphocytes differentiate into cytotoxic T lymphocytes (CTLs) which will kill virus-infected cells; (e) probiotics likewise exert immunomodulatory activities through interaction with TLRs, resulting in the initiation of downstream signaling cascades, such as NF-κβ, which induce the expression of antiviral genes (*MxA* and *OAS*) (according to [283,284,285,286]).

At the molecular level, probiotics release a wide variety of bioactive compounds into the GI tract that mediate complex interactions between the GM, epithelial cells, and the host immune system. As described by Mazziotta et al. [280], these probiotic-derived effectors include (i) proteins of diverse types, either associated with microbial surfaces or secreted extracellularly; (ii) small peptides and amino acids; (iii) bacterial DNA; and (iv) SCFAs [281]. Comparable to postbiotics, such as bacterial surface fragments, these antigens can traverse the intestinal barrier and activate immune responses [282]. Through multiple mechanisms, probiotics are able to influence host signaling pathways and regulate cytokine production, including IL-1, IL-2, IL-4, IL-6, IL-10, IL-12, IFN-γ, and TNF-α. Such modulation translates into enhanced immune functions, such as greater cytotoxic and phagocytic activity of natural killer (NK) cells and macrophages, as well as proliferation and differentiation of T and B lymphocytes, ultimately leading to stronger antibody-mediated responses [280]. Figure 3 illustrates the antiviral effects of probiotics, particularly their role in preserving intestinal barrier integrity (according to [283,284,285,286]).

Probiotics play an important role in preserving the integrity of the intestinal epithelial barrier, which is frequently disrupted during GI viral infections. Viral diarrheal diseases compromise epithelial function through multiple mechanisms, including increased secretion of water and electrolytes, enhanced paracellular permeability, and induction of epithelial cell damage and apoptosis [287]. Certain probiotic strains are capable of mitigating these detrimental effects through diverse protective strategies, thereby supporting intestinal homeostasis and barrier stability. A well-characterized example is *Lacticaseibacillus* (formerly *Lactobacillus*) *rhamnosus* GG, which secretes soluble proteins (endolysins p40 and p75) derived from cell wall biosynthesis and turnover. These proteins activate the epidermal growth factor receptor and, in turn, the PI3-K/Akt signaling pathway, thereby shielding epithelial cells from apoptosis induced by inflammatory cytokines (e.g., TNF-α, IFN-γ), oxidative stress, or chemically induced inflammation, both in vitro and in vivo [288]. In addition, low-molecular-weight peptides produced by *L. rhamnosus* GG stimulate the expression of cytoprotective heat shock proteins (HSP25 and HSP72) [289,290]. Collectively, these actions enhance epithelial resistance to apoptosis and reinforce structural integrity by upregulating tight junction proteins, including zonula occludens-1, occludin, and claudin [291].

The influence of probiotics on intestinal mucus production has been demonstrated in several studies. For instance, *L. rhamnosus* GG and *Lactiplantibacillus* (formerly *Lactobacillus*) *plantarum* 299v enhance mucin secretion both in cultured intestinal cells and in vivo in mice, primarily through the upregulation of the *MUC-2* and *MUC-3* genes. This results in increased mucus synthesis and thickening of the intestinal mucus layer [288,292]. The mucus layer, positioned above the epithelial lining, serves as a critical frontline defense against infection. It is composed of mucins and glycoproteins that interact with the GM and, in some cases, provide attachment sites and/or nutrients for enteric pathogens [293]. However, the continuous renewal of mucus and its dynamic movement along the GI tract also contribute to host protection, functioning as a physical barrier that restricts viral access to epithelial cells and facilitating viral clearance by trapping and eliminating viral particles through fecal excretion [33]. Furthermore, certain probiotics, particularly *Lactobacillus* and *Bifidobacterium* species, express surface-associated structures that enable adhesion to mucus and extracellular matrix components. These include extracellular surface layer proteins as well as specialized adhesion factors such as mucus-binding pili, which are characteristic of specific lactobacilli and bifidobacteria [294]. Another important mechanism by which probiotics exert antiviral effects is through the activity of adhesion factors. These molecules are typically bacterial cytoplasmic proteins that are translocated to the cell surface via export pathways, such as chaperone systems, glycolytic enzymes, or ABC transporters. In species including *L. plantarum*, *Lacticaseibacillus casei*, *L. reuteri*, and *Lactobacillus johnsonii*, such adhesion factors facilitate interactions with the intestinal mucosa, displaying lectin-like properties that promote bacterial colonization. Importantly, these same mechanisms can contribute to the competitive exclusion of viral pathogens by preventing their attachment to host cells. Beyond competition for adhesion sites, several probiotic strains are able to directly interact with viral particles. For instance, *L. rhamnosus* and *Bifidobacterium animalis* subsp. *lactis* Bb12 have been shown to bind RV with high efficiency [295]. Similarly, *L. casei* BL23 and *E. coli* Nissle 1917 can associate with viral particles, although the specific surface molecules mediating these interactions remain unidentified [296]. Since many viral receptors are glycosylated host proteins, it has been proposed that probiotic strains expressing surface glycoproteins or carbohydrate-rich polymers may act as receptor mimics, thereby sequestering viral particles [297]. Indeed, this has been observed in other enteric bacteria that carry sugar components structurally similar to HBGAs, enabling direct interactions with NoV [248,298].

Several studies have reported that certain probiotic strains are capable of producing antiviral compounds [299]. Among these, organic acids generated during carbohydrate fermentation exert intrinsic microbicidal effects. In vitro experiments have demonstrated that these acids can reduce the infectivity of some viruses, suggesting that an acidic environment may help limit viral replication [300]. Nevertheless, since enteric viruses have adapted to the GI environment as their natural replication niche, their tolerance to acidic conditions may restrict the overall impact of this mechanism [33]. In addition to organic acids, probiotics can stimulate the host to generate virucidal molecules. For instance, several strains, including *L. rhamnosus* GG, *L. casei* Shirota, *Lactiplantibacillus pentosus*, *L. plantarum*, and *Limosilactobacillus fermentum*, enhance host production of reactive oxygen species (ROS), such as hydrogen peroxide (H_2_O_2_) and nitric oxide (NO^−^). Depending on the strain–cell line combination, this increase may reach up to 50%, leading to a marked reduction or even complete prevention of RV-induced disruption in epithelial monolayers [301]. Notably, RV infection itself promotes chloride secretion through the viral enterotoxin NSP4, a process linked to oxidative stress. This effect can be counteracted by supernatants from the probiotic yeast *Saccharomyces boulardii*, which has been shown to suppress RV-induced ROS accumulation [302]. Another important class of probiotic-derived antiviral agents are bacteriocins. These peptide-based molecules, produced by a range of probiotic bacteria, not only provide broad health benefits but also act as potent antagonists against both bacterial pathogens and viruses. Pioneering studies have identified three principal antiviral mechanisms of bacteriocins [303]: (i) aggregation of viral particles and obstruction of host cell receptors to prevent viral entry; (ii) reduction in viral release and induction of cytopathic effects without interfering with viral penetration; and (iii) direct interaction with lipid membranes of enveloped viruses, thereby inhibiting fusion between viral and cellular membranes.

The most prominent role of probiotics in viral infections lies in their capacity to modulate host immune responses. One key mechanism involves the enhancement of secretory IgA production within the intestinal lamina propria. Elevated IgA levels contribute to viral neutralization by blocking antigen recognition and preventing viral adhesion to host cells, a process shown to be relevant in infections caused by coronaviruses [304]. Beyond IgA induction, probiotics also interact with PRRs such as TLRs, which detect pathogen-associated molecular patterns (PAMPs). This interaction activates intracellular signaling pathways, including the nuclear factor-κB (NF-κB) cascade, leading to the upregulation of antiviral defense genes [305]. Among these, *Mx1* (myxovirus resistance gene) and *OAS1a* (2′-5′-oligoadenylate synthetase 1A) are particularly important, as they mediate the production of type I and type III IFNs in lung tissue and alveolar macrophages, thereby reinforcing antiviral immunity [285]. Probiotic-driven immune modulation also shapes adaptive responses. Activated antigen-presenting cells stimulate T helper type 1 (Th1) lymphocytes, which enhance phagocytic activity and accelerate viral clearance [306,307]. Similarly, stimulation of CD8^+^ T cells promotes their differentiation into cytotoxic T lymphocytes (CTLs), specialized in eliminating virus-infected cells [284,285]. In parallel, NK cells are activated, leading to increased IFN-γ secretion and strengthening of innate antiviral defenses [308].

#### 5.1.1. In Vitro Models

In vitro models represent a valuable approach for assessing both the antiviral potential of probiotics and the underlying cellular mechanisms of their activity. Many enteric viruses, including RV and NoV, rely on specific oligosaccharides on the surface of host cells as receptors or co-receptors during the initial stages of infection. Varyukhina et al. [309] revealed that glycan-modifying bacteria, such as the probiotic *L. casei* DN114001 and the commensal *Bacteroides thetaiotaomicron*, can inhibit RV infection in the mucus-secreting human HT-29-MTX cell line. This inhibitory effect occurs via bacterial secretion of a soluble factor capable of modifying the cell surface glycans, a mechanism comparable to the activity of bovine milk-derived galactosyltransferase [309]. A distinct mechanism of anti-RV activity has been described for the probiotic yeast *S. boulardii* [302]. As previously noted, the yeast reduces RV-induced chloride secretion mediated by the viral enterotoxin NSP4 in human epithelial cells by modulating oxidative stress. Both culture supernatants of *S. boulardii* and the antioxidant N-acetylcysteine were effective in preventing chloride efflux by decreasing ROS and regulating oxidized and reduced glutathione levels. In addition, several probiotic bacterial strains have been evaluated for anti-RV activity in vitro. For instance, *Bifidobacterium longum* subsp. *infantis* CECT7210 demonstrated inhibitory effects in HT-29 and MA-104 cells [310]. Similarly, strains isolated from the feces of breast-fed infants, including *Lacticaseibacillus paracasei* CNCM I-4034, *Bifidobacterium breve* CNCM I-4035, and *L. rhamnosus* CNCM I-4036, were assessed in HT-29 cells. The supernatants from *L. rhamnosus* and *L. paracasei* exhibited strain-specific antiviral activity, whereas *B. breve* did not show any detectable inhibitory effect against RV [311].

Human NoV has historically posed challenges for in vitro cultivation. To overcome this limitation, various NoV surrogates have been employed to investigate the antiviral potential of bacterial strains. For instance, Lee et al. [312] demonstrated that both feline calicivirus and murine NoV were inactivated during the fermentation of dongchimi, a traditional Korean vegetable product, with viral titers decreasing by the end of fermentation. Similarly, feline calicivirus has been used as a human NoV surrogate to evaluate the antiviral activity of *Lactococcus lactis* subsp. *lactis* LM0230 [313]. In this study, pre-incubation of the virus either with bacterial cells or with cell-free culture supernatants led to a significant reduction in viral infection. For structural, functional, and antigenic studies, virus-like particles (VLPs) and P-particles derived from human NoV have become common surrogates. These particles have been used to assess the binding capacity of various probiotic and non-probiotic bacteria of intestinal or food origin to human NoV and to determine whether such interactions interfere with NoV-host cell binding. Notably, only the combination of *L. casei* BL23 or *E. coli* Nissle 1917 with GI.1 NoV P-particles led to reduced binding of the viral particles to HT-29 cells. In contrast, pre-incubation of either the bacteria or the P-particles with the host cells resulted in enhanced viral attachment [296]. This facilitative effect of commensal or probiotic bacteria on NoV binding has been further corroborated by studies using a human NoV cell culture system incorporating the GM [26].

In addition, probiotic candidates *Lactobacillus* spp. (probio 37 and probio 38), isolated from the porcine GI tract, have demonstrated in vitro antiviral activity against transmissible gastroenteritis coronavirus (TGC). Cell-free supernatants from both strains were capable of reducing TGC infectivity in ST cell cultures [314]. In a similar manner, the probiotic *Enterococcus faecium* NCIMB 10415 was shown to inhibit TGC infection while concurrently enhancing the viability of ST cells [315].

#### 5.1.2. Preclinical Studies

Animal models have served not only to evaluate the efficacy of probiotics but also, and more importantly, to elucidate the underlying mechanisms of their protective effects. In the context of RV infection, data have been generated across three different models, including rats, mice, and pigs. The collective findings consistently indicate that the primary mode of probiotic-mediated protection against RV-induced diarrhea involves modulation of the host immune response. Ventola et al. [316] investigated the impact of probiotic and postbiotic *L. rhamnosus* GG on simian RV SA11 infection using a newborn rat model. Although administration of the bacteria did not reduce the incidence of diarrhea, both live and inactivated forms provided measurable benefits, including mitigation of body weight loss and attenuation of colon swelling, compared to the infected control group. Notably, the newborn mouse model has provided valuable insights into the anti-RV effects of probiotics [317]. In one study, heat-inactivated *Lactobacillus gasseri* SBT2055 (postbiotic) was administered to pregnant female mice, which were subsequently orally immunized with RV SA11. After birth, pups from these dams were challenged with RV, and both diarrhea incidence and IgA levels were assessed. Results demonstrated that pups from the postbiotic-fed group exhibited reduced diarrhea, which correlated with elevated IgA production in the immunized dams that had received the probiotic [317]. In adult mice, the anti-RV potential of *B. longum* subsp. *infantis* CECT7210 was evaluated, showing delayed viral shedding at 48 h post-infection and lower antigen levels at day 7, supporting previous in vitro findings [310]. Similarly, neonatal mice infected with homologous murine RV strain EC benefited from treatment with the strains DSM 17938 and ATCC PTA 6475 of *L. reuteri*. Both strains reduced the duration of diarrhea and enhanced GM richness and diversity [318]. These protective effects were associated with suppression of pro-inflammatory mediators, including macrophage inflammatory protein-1α and IL-1β, along with reduced IL-7, IL-10, IL-12, and IFN-γ levels, and increased RV-specific antibodies. Furthermore, different dosing regimens of *L. rhamnosus* GG were tested in newborn mice to determine optimal intervention strategies. The most effective approach involved pretreatment with higher doses, which shortened diarrhea duration and reduced jejunal epithelial vacuolation, effects that were attributed to increased anti-RV IgA and IFN-γ production [319].

The gn piglet model has been extensively employed to investigate how probiotics modulate immune responses to counteract RV infections. For instance, Azevedo et al. [320] demonstrated that a combination of *Lactobacillus acidophilus* and *L. reuteri* influenced cytokine profiles in gn pigs infected with human RV, highlighting the potential of lactic acid bacteria to contribute to gut immune homeostasis. In addition, this model has been used to assess the impact of varying doses of *L. acidophilus* NCFM when administered as an adjuvant to a human RV vaccine. Interestingly, a low dose enhanced IFN-γ-producing T-cell responses while suppressing TGF-β and IL-10 production compared to a high dose, underscoring that the same probiotic strain can exert opposite immunomodulatory effects depending on the dosage, either stimulating or dampening IFN-γ or Treg cell-mediated responses [321,322]. Moreover, the combination of *L. rhamnosus* GG and *B. animalis* subsp. *lactis* Bb12, administered alone or with milk colostrum, was studied for its effect on neonatal immune responses to an oral human RV attenuated vaccine [323]. Although the experimental design was complex, results suggested that probiotics can influence neonatal antibody production in response to vaccination. In a follow-up study, the same probiotic combination was evaluated in gn piglets exposed both to the RV attenuated vaccine and to a virulent human RV strain. Notably, the immunological modulation observed in *L. rhamnosus* GG- and *B. animalis* subp. *lactis* Bb12-colonized piglets varied between the vaccine and virulent strains, emphasizing that probiotic-mediated immune effects can be strain- and context-dependent [324].

Vlasova et al. [325] demonstrated that the combination of *L. rhamnosus* GG and *B. animalis* subsp. *lactis* Bb12 also influences the innate immune response to RV in the gn piglet model. The study revealed that vaccination combined with colonization by both probiotic strains completely prevented diarrhea following virulent RV challenge. This protective effect was associated with enhanced immunomaturation, evidenced by increased frequencies of CD4^+^, SWC3a^+^, CD11R1^+^, and MHCII^+^ mononuclear cells, as well as conventional dendritic cells, in both intestinal tissues and peripheral blood after challenge. Furthermore, the gn piglet model has been instrumental in elucidating the mechanisms underlying *L. rhamnosus* GG-mediated protection against RV. Oral administration of this strain preserved ileal epithelial integrity after virulent RV infection by promoting compensatory expression of adhesion proteins α-catenin and β-catenin, tight junction proteins occludin, claudin-3, and claudin-4, and modulating the leak-associated claudin-2. *L. rhamnosus* GG also enhanced mucin production and maintained serum levels of the pro-inflammatory cytokine TGF-β [326]. In turn, this probiotic was shown to mitigate RV-induced autophagy, reducing the expression of autophagy markers ATG16L1 and Beclin-1, as well as modulating mTOR activity, thereby preventing virus-induced tissue damage [327].

#### 5.1.3. Clinical Studies

Several clinical studies have been carried out to evaluate both the safety and effectiveness of probiotic interventions in human populations, employing diverse bacterial species and strains. Due to these variations, outcomes across studies have been highly heterogeneous. For instance, an open-case controlled trial assessed the impact of *L. casei* Shirota on NoV-associated diarrhea in elderly individuals [328]. This study included 77 participants with a mean age of 84 years. The findings indicated that daily intake of milk fermented with this probiotic did not prevent NoV-induced diarrhea, although it was associated with a shorter average duration of fever.

In recent years, multiple clinical trials have investigated the impact of various probiotics on RV infections in infants. A double-blind, placebo-controlled study conducted in Brazil demonstrated the efficacy of *S. boulardii* in treating RV-associated diarrhea [329]. The trial enrolled 182 infants, 57% of whom tested positive for RV by commercial ELISA, and administration of the probiotic yeast after diarrhea onset significantly reduced its duration. In contrast, a randomized, double-blind, placebo-controlled trial in Kolkata (India) evaluated *Heyndrickxia coagulans* (formerly *Bacillus coagulans* or *Lactobacillus sporogenes*) for RV diarrhea in children, but no significant benefit was observed in the study population [330]. Similarly, a trial involving 106 infants aged 6–48 months tested *L. reuteri* DSM 17938 and failed to show improvement in RV-induced diarrhea [331]. Conversely, a prospective, randomized clinical trial assessing a combination of *S. boulardii* I-745 and *B. animalis* subsp. *lactis* B94 in 75 children demonstrated a reduction in diarrhea duration compared to controls [332]. Another probiotic formulation, BIO-THREE, which combines *Enterococcus faecalis*, *Clostridium butyricum*, and *Bacillus mesentericus*, was evaluated in a single-center, open-label controlled trial including 159 patients aged 3 months to 14 years, of whom 42 were confirmed RV-positive. Administration of BIO-THREE shortened diarrhea duration, although it did not significantly affect disease severity [333].

*L. rhamnosus* GG is among the most extensively studied probiotics across in vitro, in vivo, and clinical settings. Aggarwal et al. [334] reported in 2014 that this strain effectively reduced the duration of diarrhea in an open-case controlled study in India involving 200 children suffering from both RV and non-RV diarrheal episodes. In a subsequent randomized, double-blind, placebo-controlled trial, Sindhu et al. [335] evaluated 124 children infected with either RV or *Cryptosporidium* spp. The children receiving *L. rhamnosus* GG showed fewer recurrent diarrheal episodes and improved intestinal function following treatment. In addition, this group exhibited a significant increase in serum IgG levels post-intervention [335]. Table 1 summarizes various clinical trials investigating the use of probiotics and postbiotics for the prevention or treatment of enteric viral infections (according to [14,33,183]).

### 5.2. Fecal Microbiota Transplantation

FMT entails the transfer of microbial communities from the feces of healthy donors to recipients with a disrupted GM, with the goal of reestablishing microbial diversity and functional balance within the gut ecosystem [352]. Clinically, FMT has been applied in a broad range of conditions, including (i) recurrent or chronic infections, such as antibiotic-resistant *C. difficile*, *Helicobacter pylori*, multidrug-resistant bacterial infections, as well as certain fungal and viral infections; (ii) GI disorders, including Crohn’s disease, ulcerative colitis, chronic constipation, celiac disease, IBS, colorectal cancer, and chronic pouchitis; (iii) metabolic and autoimmune diseases, such as MASLD, metabolic syndrome, type 2 diabetes, obesity, fibromyalgia, refractory melanoma, and cardiovascular inflammation; (iv) neurodegenerative disorders, including Alzheimer’s disease, Parkinson’s disease, and multiple sclerosis; and (v) mental health conditions, such as anxiety, depression, bipolar disorder, and eating disorders [353,354,355,356,357,358,359]. Despite its use in these areas, the potential of FMT for treating viral GI infections remains largely unexplored.

Barberio et al. [360] described the case of a 68-year-old immunocompromised woman who experienced persistent, severe chronic diarrhea in the absence of typical GI or systemic symptoms such as nausea, vomiting, or fever. Laboratory testing of her stool samples was negative for bacterial and parasitic pathogens but positive for NoV. Alternative treatments, including antibiotics and probiotics, failed to resolve her symptoms, and NoV detection in stools persisted. The patient subsequently underwent FMT via colonoscopy, which led to complete resolution of diarrhea, and NoV tests remained negative for at least five months post-procedure. FMT has also been applied in the management of colitis associated with HCMV infection in immunosuppressed pediatric patients with ulcerative colitis [361]. In this study, eight children received FMT via nasogastric tube for five consecutive days over two weeks. By the sixth week, colonic CMV DNA PCR testing was negative in 7 of 8 patients after a total of 10 infusions. One patient required 20 infusions to achieve CMV clearance from colonic biopsies. Clinically, three patients showed a response, while another three achieved remissions.

Serrano-Villar et al. [362] investigated the effects of FMT in 30 HIV-infected individuals receiving antiretroviral therapy and found the procedure to be safe, with no serious adverse events reported. FMT led to partial restoration of gut microbial balance, attenuating HIV-associated dysbiosis. Specifically, the treatment induced notable changes in GM composition, including significant increases in alpha diversity, along with a mild and transient engraftment of the donor GM during the intervention period. Furthermore, the FMT-treated group showed a significant reduction in intestinal fatty acid-binding protein, a biomarker of gut epithelial damage and an independent predictor of mortality.

More recently, Bespyatykh et al. [363] applied FMT to a 2.5-year-old patient with immunodeficiency who had undergone allogeneic hematopoietic stem cell transplantation and was suffering from GI symptoms due to secondary infections with astrovirus and *C. difficile*. Following two FMT sessions, the patient’s intestinal symptoms resolved, and subsequent tests for astrovirus RNA and clostridial toxins were negative. Analysis of fecal metabolites revealed increased levels of cholic acid and, notably, deoxycholic acid, along with their glycine- and taurine-conjugated forms. Moreover, acetic acid levels increased while propionic acid decreased, reflecting the recovery of the functional capacity of the intestinal microbiota.

FMT has also been considered as a potential therapeutic strategy for chronic HBV infection, due to its capacity to restore GM homeostasis [364]. Ren et al. [365] conducted a clinical trial in HBeAg-positive chronic HBV patients receiving ongoing entecavir or tenofovir therapy and found that FMT promoted HBeAg clearance in a subset of patients who had persistent positivity despite long-term antiviral treatment. These findings suggest that FMT may serve as an adjunctive intervention to modulate GM in chronic HBV. Supporting this notion, Yang et al. [366] demonstrated that asymptomatic HBV carriers exhibit altered GM, indicating that GM modulation may contribute to maintaining viral tolerance without overt disease. In addition, a pilot study by Chauhan et al. [367] involving 12 HBV-infected patients administered six cycles of FMT via the nasoduodenal route at four-week intervals alongside standard antiviral therapy reported HBeAg clearance in 16.7% of participants, whereas none achieved clearance with antiviral therapy alone. The authors concluded that FMT appears to be safe and shows potential for enhancing viral suppression and HBeAg clearance in HBeAg-positive chronic hepatitis B, although larger randomized controlled trials are necessary to confirm these observations.

Interestingly, Ebrahimi et al. [368] performed a systematic review encompassing eight studies with a total of 196 participants affected by various enteric viral infections, including HIV, SARS-CoV-2 co-infected with *C. difficile*, HCMV, and HBV. Although the efficacy of FMT differed across studies and viral types, the review concluded that FMT represents a potentially safe and promising therapeutic approach for certain viral infections. Nevertheless, the observed variability in outcomes and the limited sample sizes highlight the need for additional well-designed clinical trials to better assess the effectiveness of FMT in viral diseases. Table 2 summarizes several clinical trials investigating the use of FMT in the management of various GI viral infections.

## 6. Discussion

Disruptions in GM balance, caused by diverse factors such as antibiotic usage, dietary changes, or alterations in host immune function, can increase susceptibility to pathogenic microorganisms, thereby influencing disease severity and overall host health [370]. In turn, modifications in the GM may also confer resistance against certain pathogens, including enteric viruses [222,371]. Viral infection begins with the entry of the virus into the host, where it encounters the skin or mucosal surfaces densely colonized by commensal microorganisms. This establishes a complex interplay between invading viruses, resident microbiota, and the host immune system, which can perturb the delicate homeostatic balance. In this context, clinical factors, such as pre-existing health conditions, can constitute variables that influence viral disease outcomes [372].

### 6.1. Interactions Between the Gut Microbiome and Enteric Viral Infections

Research examining the interplay between viruses and bacteria and its impact on the GM remains limited and often yields conflicting results. Preclinical mouse studies indicate that mixed viral–bacterial infections lead to a more pronounced disruption of GM composition compared to infections with RV or NoV alone [195,373]. These findings suggest that factors beyond the viral pathogens themselves contribute to disease severity, with the GM playing a central role in shaping immune responses and influencing the pathogenesis of GI infections. Thus, addressing questions that still remain unanswered will require larger follow-up studies to disentangle the contributions of variables such as viral genotype, replication levels, coinfections with other pathogens, host genetics, and additional environmental or microbial factors. Much of the existing literature remains correlative, leaving causal relationships poorly defined. For instance, enteric viral infections themselves can modulate GM composition. Accordingly, animal models and in vitro systems represent valuable approaches for probing these causal links and the underlying molecular mechanisms [374,375]. Moreover, a deeper understanding of how GM-derived secondary metabolites activate host immunity is needed, including the role of bacterial extracellular vesicles [376]. These vesicles have been proposed as communication mediators both among bacteria and between bacteria and host cells, highlighting their potential significance in infectious disease processes [377].

Considering all these recent findings, it is evident that further investigation is needed to clarify how the GM influences enteric viral infections. Several key questions remain regarding the mechanisms by which bacteria modulate viral replication and pathogenesis. First, it is critical to identify the specific bacterial taxa that enhance viral infection. For instance, studies indicate that poliovirus and reovirus exhibit distinct binding preferences for particular bacterial species and cell wall components. In this respect, elucidating the molecular basis of these affinities could reveal novel therapeutic targets. Second, biological sex may modulate these microbial interactions. Differences in GM composition between males and females are well documented and have been shown to affect host immune responses [378]. Notably, recent evidence indicates that intestinal replication of coxsackievirus B3 is sex-dependent [379]. Given that bacteria interact with coxsackievirus B3, sex-specific microbiota could potentially influence viral replication through both direct and indirect mechanisms. Third, the contributions of non-bacterial components of the GM to enteric viral infections remain largely unexplored. While current research has predominantly focused on bacterial effects, the roles of the gut mycobiome and virome in modulating viral infections are not yet established, despite their demonstrated influence on immune responses and IBD [380,381]. Thus, expanding research to include these microbial communities will be essential for a more comprehensive understanding of GM–virus interactions.

An important limitation of this review is the lack of consideration of host genetic factors in modulating interactions between the GM and enteric viral infections. Host genetics, particularly HBGAs and secretor status, have been linked to susceptibility to NoV and RV infections [382,383]. Secretor status is determined by the *FUT2* gene, which encodes an enzyme essential for the synthesis of glycans in saliva and intestinal mucus. These glycans are also associated with the composition of the GM [384]. Certain NoV strains exploit these glycans as attachment sites to initiate infection [385]. Individuals homozygous for nonfunctional *FUT2* alleles are classified as non-secretors and are highly resistant to symptomatic NoV infection, as the absence of functional glycans limits viral binding to intestinal receptors, thereby preventing cell entry and infection [385]. Interestingly, Rodriguez-Diaz et al. [383] observed that higher IgA titers against NoV and RV correlated with secretor-positive status. However, no statistically significant differences were detected among different *FUT2* genotypes. Furthermore, the study found that the relative abundance of bacterial genera such as *Faecalibacterium* and *Ruminococcus* was associated with lower IgA responses against NoV and RV, suggesting a complex interplay between host genetics, GM composition, and viral susceptibility. Despite these insights, the role of secretor status in NoV infection remains debated [14]. Future studies employing human enteroid models, which recapitulate the intestinal epithelium, could help clarify these relationships [386]. Thus, generating libraries of enteroids from individuals with diverse *FUT2*, *FUT3*, and *ABO* genotypes may provide valuable data on how secretor status, Lewis antigens, and other HBGAs influence susceptibility to NoV infection [387].

### 6.2. Microbiota-Based Interventions

The notion of “microbiota modulation” is emerging as a potential antiviral strategy aimed at preventing viral infections and mitigating severe outcomes following infection. Daily lifestyle interventions may play a key role in shaping the GM. For instance, moderate physical activity has been associated with multiple health benefits, including reduced systemic inflammation, enhanced intestinal barrier function, and improved body composition [388]. Moreover, interventions targeting mental well-being, such as stress reduction programs and measures to maintain oral health, have been shown to promote a balanced intestinal environment and preserve GM homeostasis [389,390,391]. In the context of mental health, it is also relevant to note that viral infections may contribute to neuropsychiatric disorders, such as schizophrenia, through immune system modulation and alterations in brain development [392]. Collectively, these insights emphasize the need for continued research to elucidate the complex interactions between lifestyle, the GM, viral exposures, and both immune and mental health.

When considering probiotics as a strategy to control enteric viral infections, it is important to note that study designs remain highly heterogeneous, and no standardized approach has yet been established for testing different probiotic strains. In addition, most published studies were single-center trials with relatively small patient cohorts. The European Society for Pediatric Gastroenterology, Hepatology, and Nutrition, together with the European Society for Pediatric Infectious Diseases, recently released their “Evidence-Based Guidelines for the Management of Acute Gastroenteritis in Children in Europe”. In these guidelines, only two probiotic species (*L. rhamnosus* GG and *S. boulardii*) are strongly recommended, and always in conjunction with oral rehydration therapy. However, the recommendation is qualified by “moderate to low quality of evidence” regarding their effectiveness in reducing symptom duration and severity [393], highlighting the need for more rigorous research to substantiate probiotic use against viral diarrhea in humans. Furthermore, several *Bifidobacterium* strains have demonstrated protective effects against RV in vitro, in animal models, and in pediatric populations [310,345]. This antiviral effect is likely mediated by small peptides derived from *Bifidobacterium* metabolism of milk casein [394]. Although the precise mechanisms through which these viral interactions occur remain incompletely understood, they may involve enhanced goblet cell proliferation and increased mucin production, contributing to improved intestinal barrier function [395].

FMT, another microbial-based tool examined in the present review, has emerged as a potential therapeutic approach for viral diseases, with reported benefits including viral clearance, reduction in inflammation, and improvement of clinical outcomes. However, the current evidence is limited, and FMT efficacy appears to vary depending on the viral pathogen [368]. Given the heterogeneous responses and possible effects on gut health, caution is advised when considering FMT for viral infections. Large-scale, well-controlled clinical trials are necessary to define the precise role of FMT in viral disease management and to develop standardized treatment protocols for clinical practice. Building on these considerations, future research should incorporate standardized adverse event reporting, clearly defined and validated endpoints, larger patient cohorts, and extended follow-up periods to generate more robust evidence regarding both safety and effectiveness [396]. In addition, studies should investigate how variations in FMT protocols, such as route of administration, frequency and volume of fecal matter transplanted, use of adjunctive therapies to enhance engraftment, and donor characteristics including age, sex, diet, and lifestyle, affect clinical outcomes. This information will be pivotal to optimize FMT as a personalized therapeutic strategy for viral diseases. Therefore, future microbial-based interventions, including new probiotic candidates and adapted FMT strategies, must be investigated to evaluate their effectiveness and ensure safety [397].

## 7. Conclusions

The understanding of how the GM influences human health and disease remains incomplete. The complexity of individual microbiomes, coupled with the extensive variability observed across human populations, presents significant challenges to mechanistic studies. Recent research has increasingly focused on the direct interactions between commensal bacteria and human enteric viruses, aiming to clarify the specificity of these interactions and their consequences for viral infection. In this respect, future investigations should prioritize elucidating the effects of microbial metabolites on both viral particles and host immune responses, encompassing local intestinal immunity as well as systemic innate and adaptive responses. Although there is growing interest in integrating GM analyses into studies of host antiviral immunity, the influence of endogenous microbial factors on frontline defense mechanisms and the formation of protective immunological memory remains insufficiently understood. Therefore, a deeper mechanistic insight into GM–host–virus interplay is essential to advance the development and clinical application of innovative therapeutics, including engineered probiotics, biotherapeutics, and phage-based interventions. Moreover, precision microbiome therapeutics will be critical not only for viral infections but also for broader GI diseases. In summary, the role of the GM in modulating enteric viral infections represents a promising area of future investigation. Advancing this research is pivotal to fully understand the environmental and microbial determinants that shape viral pathogenesis in the host. Ultimately, the GM represents a dynamic factor that shapes host susceptibility and response to enteric viral infections. Integrating microbiome insights with virology and immunology could enable predictive and personalized strategies for prevention and treatment, positioning the GM as both a therapeutic target and a biomarker for disease outcomes. Since current evidence is still limited, future studies should focus on addressing gaps in mechanisms, host factors, and clinical validation.

## Figures and Tables

**Table 1:** Clinical trials on probiotics and postbiotics use against enteric viral infections.

Agents	Target Virus	Results	Reference
*Bifidobacterium longum* strain BORI + *Lactobacillus acidophilus* strain AD031	RV	The duration of diarrhea among patients was significantly shorter in the probiotics group than in the placebo group. Symptoms such as duration of fever, frequency of diarrhea, and frequency of vomiting tended to be ameliorated by the probiotic treatment. However, differences were not statistically significant between the two groups.	[28]
*Lactiplantibacillus plantarum* strain LRCC5310	RV	The probiotic improved clinical symptoms, including diarrhea and Vesikari score, and also inhibited viral proliferation in RV gastroenteritis.	[29]
*Lacticaseibacillus casei* strain Shirota	NoV	Continuous intake of the probiotic could positively contribute to the alleviation of fever caused by NoV gastroenteritis by correcting the dysbiosis of the GM typical in the elderly, although probiotic could not protect from the disease.	[328]
*Saccharomyces boulardii*	RV	The yeast probiotic reduced diarrhea duration in infants compared to placebo group.	[329]
*Heyndrickxia coagulans*	RV	Differences in recovery rate, duration, frequency, and volume of diarrhea were not significant between both groups (probiotic vs. placebo).	[330]
*Limosilactobacillus reuteri* strain DSM 17938	RV	Probiotic did not significantly affect the risk of developing nosocomial diarrhea or RV infection. There was also no difference between the probiotic and placebo groups for any of the other secondary outcomes (i.e., incidence of RV infection, incidence of diarrhea, duration of diarrhea, incidence of recurrent diarrhea, incidence of chronic diarrhea, length of hospital stay in days, and frequency of need for rehydration).	[331]
*S. boulardii* strain I-745 + *Bifidobacterium animalis* subsp. *lactis* strain B94	RV	The duration of diarrhea was shorter in the group with oral rehydration therapy and rapid refeeding via a normal diet with the probiotic mixture than in the group with only oral rehydration therapy and rapid refeeding via a normal diet.	[332]
*Enterococcus faecalis* strain T110 + *Clostridium butyricum* strain TO-A+ *Bacillus mesentericus* strain TO-A	RV and *Salmonella*	Seven-day BIO-THREE administration demonstrated high efficacy and safety in infants and children with severe gastroenteritis. The incidence of severe gastroenteritis was significantly reduced in the RV origin and BIO-THREE intervention groups.	[333]
*Lacticaseibacillus rhamnosus* strain GG	RV	The results showed that the use of the probiotic in children with acute diarrhea resulted in shorter duration and faster improvement in stool consistency compared to the control group. These benefits were seen irrespective of RV positivity in stool tests.	[334]
*L. rhamnosus* strain GG	RV or *Cryptosporidium* spp.	Probiotic strain had a positive immunomodulatory effect and may be useful in decreasing repeated episodes of RV diarrhea. Significant increased IgG levels post-intervention.	[335]
*L. rhamnosus* strain GG	RV	Probiotic supplementation did not decrease the frequency and duration of diarrhea and vomiting in children with acute watery diarrhea, and did not reduce hospital stay in these patients.	[336]
*S. boulardii* strain CNCM I-3799	RV	A significantly shorter duration of hospitalization was achieved in the intervention group, but no significant difference was obtained for fever and vomiting between intervention and control groups.	[337]
*L. acidophilus*, *Lactobacillus delbrueckii* subsp. *bulgaricus*, *L. plantarum*, *L. casei*, *Bifidobacterium breve*, *B. longum*, *Bifidobacterium longum* subsp. *infantis*, and *Streptococcus thermophilus*	RV	The administration of the probiotic cocktail produced a diminished diarrhea duration and diminished number of defecation times.	[338]
*L. rhamnosus* strain R0011 + *Lactobacillus helveticus* strain R0052	RV	No beneficial virus-specific clinical effects associated with the administration of a 5-day course of a *L. helveticus*/*L. rhamnosus* combination probiotic, for children with AGE. Similarly, probiotic administration did not result in more rapid clearance of viral pathogens from stool specimens compared with placebo.	[339]
*L. casei* strain Shirota	CMV and EBV	Regular ingestion of the probiotic reduced plasma CMV and EBV antibody titers, an effect that can be interpreted as a benefit to overall immune status.	[340]
*L. acidophilus*, *L. rhamnosus*, *B. longum* and *S. boulardii*	RV	The administration of the probiotic mixture decreased the duration of diarrhea compared to oral rehydration solution alone. This decrease was significant only for the administration of *S. boulardii* single probiotic, which also decreased the duration of fever.	[341]
*B. animalis* subsp. *lactis* strain Bb12	RV and Poliovirus	The probiotic increased the anti-RV- and anti-poliovirus-specific IgA in infants.	[342]
*L. casei* strain Shirota	HIV	After probiotic ingestion, peripheral CD4^+^ T-cell, Th17, and Th2 counts significantly increased in HIV-infected groups. Conversely, CD8^+^ cells decreased in HIV(+) patients, and plasma HIV load decreased slightly but significantly among HIV(+) patients.	[343]
*Bifidobacterium adolescentis strain SPM1605*	Coxsackievirus B3	The probiotic demonstrated antiviral activity by affecting the IFN-mediated antiviral response (*MxA* gene expression).	[344]
*B. longum*, *B. animalis* subsp. *lactis*, *L. acidophilus*, *L. rhamnosus*, *L. plantarum*, and *Pediococcus pentosaceus*	RV	Of the tested probiotic strains, *B. longum* and *L. acidophilus* showed the greatest inhibitory effects. These probiotics significantly shortened the duration of diarrhea compared with a placebo, and did not induce any adverse effects.	[345]
*B. animalis* subsp. *lactis* strain Bb12 + *S. thermophilus* strain TH4	RV	The duration of the diarrhea was not influenced by the intake of probiotics. However, a decrease in RV shedding was observed in infants fed with the probiotic mixture.	[346]
Postbiotic: heat-inactivated *E. faecalis* strain FK-23	HCV	Significant decreases in mean ALT levels were observed at 3 months as compared to the initial level and persisted up to 36 months. Decrease in AST was detected after 9 months of postbiotic therapy compared to the initial level.	[347]
*L. rhamnosus* strain GG	RV	Probiotic did not change the duration of diarrhea, total diarrhea stools, or diarrhea score compared to placebo. There was a significant difference in diarrhea frequency on day 2 between probiotic and placebo groups.	[348]
*Lacticaseibacillus paracasei* strain ST11	RV	The strain ST11 of *L. paracasei* had a clinically significant benefit in the management of children with nonrotavirus-induced diarrhea, but it was ineffective in those with RV diarrhea.	[349]
*L. rhamnosus* strain GG	RV	Administration of the probiotic shortened the duration of RV diarrhea in children but not of diarrhea of any etiology. Intervention shortened the time of intravenous rehydration.	[350]
*C. butyricum* strain CGMCC0313-1 + *B. longum* subsp. *infantis* strain CGMCC0313-2	HBV	Reduction in venous ammonia and improvements in the parameters of the intestinal mucosal barrier were achieved.	[351]

RV: rotavirus; NoV: norovirus; CMV: cytomegalovirus; EBV: Epstein–Barr virus; HIV: human immunodeficiency virus; HCV: hepatitis C virus; HBV: hepatitis B virus; ALT: alanine aminotransferase; AST: aspartate transaminase; GM: gut microbiome; IFN: interferon.

**Table 2:** Clinical trials on FMT use against enteric viral infections.

Intervention	Procedure	Results	Reference
Clinical case. *n* = 1 (68-year-old woman) with severe chronic diarrhea and positive for NoV infection	Colonoscopy (250 mL of fresh fecal material from a donor).	NoV tests were performed from 5 days to 5 months following FMT. All of them were negative for virus detection. No significant adverse events of clinical interest were observed attributable to FMT. An important change in the recipient’s GM was recorded.	[360]
Clinical trial. *n* = 8 children with mild to severe ulcerative colitis and positive for CMV infection	Nasogastric tube (50–100 mL FMT by 5 days in each 2 weeks).	Negative CMV test was recorded in 7 from 8 patients at the 6th week following FMT. Clinical remission was obtained in 3 from 8 children. No serious adverse effects were observed.	[361]
Randomized controlled trial. *n* = 30 HIV-infected subjects on antiretroviral therapy	Fecal microbiota capsules for 8 weeks.	FMT was safe, not related to severe adverse events, and attenuated HIV-associated dysbiosis. FMT elicited changes in GM structure, including significant increases in alpha diversity. A significant amelioration was noted in the FMT group in intestinal fatty acid-binding protein, which is a biomarker of intestinal damage	[362]
Clinical case. *n* = 1 (2.5-year-old with immunodeficiency who had undergone allogeneic hematopoietic stem cell transplantation, suffering from GI symptoms due to secondary infections with astrovirus and *C. difficile*).	FMT from the father’s feces.	FMT promoted intestinal recolonization and eradication of GI symptoms in the patient. After two FMT procedures, the tests for the astrovirus RNA and clostridial toxins were negative.	[363]
Open-label pilot trial. *n* = 18 chronic HBV patients who remained persistently positive for HBeAg following positive receiving entecavir or tenofovir therapy for more 3 years.	FMT via gastroscope (1 to 7 treatments for 4 weeks).	FMT promoted HBeAg clearance in a subset of patients (*n* = 5) who had persistent positivity despite long-term antiviral treatment in comparison with 13 control patients. In addition, FMT could serve as an adjunctive intervention to modulate GM in chronic HBV.	[365]
Clinical trial. *n* = 12 patients with hepatitis B as intervention group, and *n* = 15 HBeAg-positive patients who were on oral antivirals for > 1 year were considered as control-AVT.	Six cycles of FMT via gastroscope (nasoduodenal route) at 4 weekly intervals.	In the FMT arm, 2 from 12 patients had HBeAg clearance in comparison to none in the AVT arm. None of the patients in either arm had HBsAg loss. The FMT was tolerated well, although 6 patients reported one or more minor adverse events.	[367]
A retrospective, single-center study. *n* = 86 patients (46 co-infected with COVID-19 and *C. difficile*) receiving antibiotics and FMT, and 40 co-infected patients who received antibiotics only (control group).	Colonoscopy (filtered solution composed by 50 g feces from first- and second-degree donors in 500 mL of saline solution).	A significant decrease in inflammatory syndrome was recorded in co-infected patients receiving FMT in addition to antibiotics, with a lower relapse rate and mitigation of cramping and abdominal pain. FMT improved patients’ quality of life and inflammatory syndrome.	[369]

NoV: norovirus; CMV: cytomegalovirus; HIV: human immunodeficiency virus; HBeAg: HBV e-antigen; AVT: antiviral therapy; GM: gut microbiome; GI: gastrointestinal; FMT: fecal microbiota transplantation.

## Data Availability

No new data were created or analyzed in this study. Data sharing is not applicable to this article.

## Abbreviations

The following abbreviations are used in this manuscript:

ACE2	Angiotensin-converting enzyme 2
AGE	Acute viral gastroenteritis
AHE	Acute hepatitis E
BAs	Bile acids
CAR	Coxsackievirus and adenovirus receptor
CI	Confidence interval
COVID-19	Coronavirus disease 2019
EBV	Epstein–Barr virus
ELISA	Enzyme-linked immunosorbent assay
FMT	Fecal microbiota transplantation
FXR	Farnesoid X receptor
GALT	Gut-associated lymphoid tissues
GF	Germ-free
GI	Gastrointestinal
GM	Gut microbiome
gn	Gnotobiotic
GWAS	Genome-wide association studies
HAdV	Human adenoviruses
HAV	Hepatitis A virus
HBeAg	HBV e-antigen
HBGA-like	Histo-blood group antigen-like
HBV	Hepatitis B virus
HCC	Hepatocellular carcinoma
HCV	Hepatitis C virus
HCMV	*Human cytomegalovirus*
HDAC	Histone deacetylase
HDV	Hepatitis D virus
HEV	Hepatitis E virus
HFMD	Hand, foot and mouth disease
HHV 8	Human herpesvirus 8
HIV	Human immunodeficiency virus
HPV	Human papillomavirus
HSV	Herpes simplex virus
IBS	Irritable bowel syndrome
IBD	Inflammatory bowel disease
IECs	Intestinal epithelial cells
IFNs	Interferons
IM	Infectious mononucleosis
KS	Kaposi’s sarcoma
LPLs	Lamina propria lymphocytes
LPS	Lipopolysaccharide
MASLD	Metabolic dysfunction-associated steatotic liver disease
MERS-CoV	Middle East respiratory syndrome-related coronavirus
MNPs	Mononuclear phagocytes
MR	Mendelian randomization
NF-κB	Nuclear factor-κB
NoV	*Norovirus*
OR	Odds ratio
PAMPs	Pathogen-associated molecular patterns
PCR	Polymerase chain reaction
PGN	Peptidoglycan
PRRs	Pattern recognition receptors
RCT	Randomized controlled trial
ROS	Reactive oxygen species
RT-PCR	Real time PCR
RV	*Rotavirus*
SARS-CoV-2	Severe acute respiratory syndrome coronavirus 2
SCFAs	Short-chain fatty acids
STAT1	Signal transducer and activator of transcription 1
TGC	Transmissible gastroenteritis coronavirus
TGEV	Transmissible gastroenteritis virus
TLRs	Toll-like receptors
Treg cells	Regulatory T cells
VIGD	Viral infectious gastrointestinal diseases
VZV	Varicella-zoster virus
